# Transcriptome analysis identifies *Bacillus anthracis* genes that respond to CO_2_ through an AtxA-dependent mechanism

**DOI:** 10.1186/1471-2164-15-229

**Published:** 2014-03-25

**Authors:** Andrew T McKenzie, Andrei P Pomerantsev, Inka Sastalla, Craig Martens, Stacy M Ricklefs, Kimmo Virtaneva, Sarah Anzick, Stephen F Porcella, Stephen H Leppla

**Affiliations:** 1 Microbial Pathogenesis Section, Laboratory of Parasitic Diseases, National Institute of Allergy and Infectious Diseases, Bethesda, MD 20892, USA; 2 Genomics Unit, Research Technologies Section, Rocky Mountain Laboratories, National Institute of Allergy and Infectious Diseases, Hamilton, MT 59840, USA; 3 National Institutes of Health, 33 North Drive, Building 33, Room 1W20B, Bethesda, MD 20892, USA

**Keywords:** *Bacillus anthracis*, pXO1, Illumina, RNA sequencing, AtxA, Toxin production, Small RNA, Gene-environment interaction

## Abstract

**Background:**

Upon infection of a mammalian host, *Bacillus anthracis* responds to host cues, and particularly to elevated temperature (37°C) and bicarbonate/CO_2_ concentrations, with increased expression of virulence factors that include the anthrax toxins and extracellular capsular layer. This response requires the presence of the pXO1 virulence plasmid-encoded pleiotropic regulator AtxA. To better understand the genetic basis of this response, we utilized a controlled *in vitro* system and Next Generation sequencing to determine and compare RNA expression profiles of the parental strain and an isogenic AtxA-deficient strain in a 2 × 2 factorial design with growth environments containing or lacking carbon dioxide.

**Results:**

We found 15 pXO1-encoded genes and 3 chromosomal genes that were strongly regulated by the separate or synergistic actions of AtxA and carbon dioxide. The majority of the regulated genes responded to both AtxA and carbon dioxide rather than to just one of these factors. Interestingly, we identified two previously unrecognized small RNAs that are highly expressed under physiological carbon dioxide concentrations in an AtxA-dependent manner. Expression levels of the two small RNAs were found to be higher than that of any other gene differentially expressed in response to these conditions. Secondary structure and small RNA-mRNA binding predictions for the two small RNAs suggest that they may perform important functions in regulating *B. anthracis* virulence.

**Conclusions:**

A majority of genes on the virulence plasmid pXO1 that are regulated by the presence of either CO_2_ or AtxA separately are also regulated synergistically in the presence of both. These results also elucidate novel pXO1-encoded small RNAs that are associated with virulence conditions.

## Background

*Bacillus anthracis* is a spore-forming, gram-positive bacterium that is the causative agent of anthrax. The major factors responsible for its pathogenesis are encoded on two virulence plasmids, pXO1 and pXO2 [1-4]. pXO1 contains the genes coding for the three secreted toxin proteins, protective antigen (PA), lethal factor (LF), and edema factor (EF). PA binds to cellular receptors, forms a complex with both EF (forming edema toxin) and LF (forming lethal toxin), and acts as a translocase [4,5]. pXO2 encodes for a γ-linked poly-D-glutamic acid capsule, which protects the bacterium from host phagocytosis [6]. The production of these virulence factors is highly dependent on an environment containing carbon dioxide and bicarbonate (CO_2_/HCO_3_, henceforth CO_2_). The relative concentrations of CO_2_ and bicarbonate are regulated enzymatically by carbonic anhydrase, as well as many other non-enzyme factors, and are not always perfectly correlated. Given that the concentration of CO_2_ is often higher in mammalian tissues than other environments, CO_2_ is thought to be an important signal for turning on virulence gene expression [7]. This CO_2_-induced response is mediated, at least in part, at the transcriptional level [8-11]. However, CO_2_ affects the transcription of many genes in other species of the *B. cereus* group; thus, it is unlikely that all of these genes are involved in anthrax-specific pathogenesis [12]. Parsing genes into pathogenic and non-pathogenic anthrax-specific subsets would help in identifying molecular virulence pathways in *B. anthracis*, which are not fully established [7,11,13].

The best characterized pleiotropic regulator of virulence-related genes in *B. anthracis* is AtxA (for Anthrax to*x*in activator) [14,15]. AtxA is a transcriptional regulator containing two DNA binding domains in the N-terminal region [11]. At the transcriptional level, production of *atxA* mRNA is upregulated by temperatures close to 37°C [16], repressed by the transition-state regulator AbrB [17,18], and impacted by the cellular redox potential [19], but these changes are all quite modest. At the post-translational level, AtxA is regulated via the phosphorylation of two histidine residues [20] and an unknown mechanism requiring the presence of the global regulator CodY [21,22]. Another post-translational regulatory effect is that elevated CO_2_ increases the ratio of dimeric to monomeric AtxA by approximately two-fold [23]. AtxA also is sufficient to cause CO_2_-related induction of PA in *B. anthracis*[24], can interact with the pXO2-regulators AcpA and AcpB to upregulate the transcription of the capsule operon [25], and AtxA can engage in regulatory cross-talk with genes on the chromosome [26]. Although AtxA is known to have a strong synergistic relationship with CO_2_[15], its own transcription has been found to increase only slightly, if at all, in the presence of CO_2_[11,12,16], indicating that its regulatory function is linked to activation rather than to changes in its mRNA expression.

Several previous studies analyzed differential gene expression of *B. anthracis* and *B. cereus* strains and mutants, in some cases exploring the effects of CO_2_. Two of these studies used microarrays based on annotated genes, and did not examine intergenic regions [12,27]. One study employed RNA sequencing, but did not examine an AtxA mutant nor attempt to assess transcripts from intergenic regions [28]. Thus, this study is unique in that we analyzed transcriptional variance across the combination of CO_2_, ambient air conditions, and wildtype (WT) and AtxA-deficient (ΔAtxA) strains using Next Generation RNA Sequencing (RNA-seq), which allows for the discovery of novel, un-annotated RNAs, small RNAs and the context of expression of adjacent chromosomal or plasmid-encoded open reading frames.

## Methods

### Strains and plasmids

Additional file 1: Table S1 lists plasmids, strains, and primers used in this study. *B. anthracis* Ames 35 is a pXO1^+^, pXO2^−^ strain closely related to the Ames Ancestor strain, which is now considered the *B. anthracis* reference genome strain (Accessions NC_007530 and NC_007322 for chromosome and pXO1 sequences, respectively). All locus tags used in this study refer to the Ames Ancestor strain, and we omitted the underline for chromosomal genes (*e.g*., GBAA0887 is used for GBAA_0887).

### Generation of a markerless deletion strain for AtxA

We followed the Cre-loxP deletion strategy for *B. anthracis* described previously [29]. Briefly, the thermosensitive plasmid pSC was used to construct separate vectors containing DNA fragments amplified from regions upstream and downstream of the *atxA* gene (see Additional file 1: Table S1 for a list of all primers and plasmids used). We transformed Ames 35 with the pSC vector containing the *atxA* upstream region, selected for a single crossover event by temperature restricted growth, and excised the inserted plasmid by introducing a plasmid expressing Cre recombinase, thereby leaving a single *loxP* site. The process was repeated with the pSC vector containing the *atxA* downstream region, with the resulting strain containing a single *loxP* site, thus replacing *atxA,* which was confirmed by Sanger sequencing.

The mutation was complemented by transformation of the Ames 35 ΔAtxA strain with plasmid pIU68 [14], which contains the *atxA* gene and its endogenous promoter region. Expression of PA was assessed in the Ames 35, ΔAtxA, and the complemented strains grown in 16 mL of NBY medium (0.8% (w/v) nutrient broth, 0.3% (w/v) yeast extract, and 0.5% (w/v) glucose) at 37°C, 225 rpm in air. For growth with CO_2_, the medium was supplemented with 0.8% (w/v) NaHCO_3_ and the air was supplemented with 15% CO_2_. Five μg ml^−1^ tetracycline was added for the strain harboring pIU68. Following growth to similar Optical Density at 600 nm (OD600) values (Ames 35 = 1.9, ΔAtxA = 1.6, and Ames 35 ΔAtxA + pIU68 = 1.5), culture supernatants were supplemented with EDTA to 5 mM and concentrated 13-fold using a 50,000 Da cut-off membrane (Sartorius, Bohemia, NY). Sample separation and Western blotting were performed using standard protocols. PA was detected using a 1:2500 dilution of anti-PA rabbit serum #5308 [30] as the primary antibody and polyclonal goat anti-rabbit IR800 conjugate (Rockland) as the secondary antibody, followed by imaging using an Odyssey infrared scanner (Li-Cor, Lincoln, NE).

### RNA isolation

Ten ml of bacterial culture grown to an average OD600 of 2.1 (Additional file 1: Figure S1) in NBY with or without CO_2,_ as indicated above, were pelleted by centrifugation at 3,500 rpm for 10 min and bacteria were lysed by addition of 1 ml of room temperature Trizol (Life Technologies, Carlsbad, CA). The mixture was then transferred to FastRNA vials containing glass beads (Qbiogene, Irvine, CA), and homogenized for 40 sec. Samples were centrifuged for 1 min at 4°C and 13,000 g, and the aqueous phases were transferred to tubes containing 200 μl of 1-bromo-3-chloropropane (Sigma-Aldrich, St. Louis, MO). The mixture was vortexed for 15 sec, heated for 20 min at 65°C, and centrifuged for 15 min at 4°C and 12,000 g. The aqueous phase was passed through a QiaShredder column (Qiagen, Valencia, CA) to eliminate genomic DNA. Equal volumes of both RLT buffer (Qiagen) + 1% β-mercaptoethanol and 70% ethanol were added to the samples. Total RNA was purified using the All Prep DNA/RNA kit (Qiagen) following the manufacturer’s protocol, and employing the optional on-column DNase I treatment. The quality of each RNA sample was assessed using a Bioanalyzer (Agilent, Santa Clara, CA) with a quality requirement of an integrity number > 8.0 for subsequent sequencing. RNA from two biological replicates was collected for each strain and condition.

### Depletion of ribosomal RNA

Non-ribosomal RNAs were enriched using the MicrobEnrich™ kit (Life Technologies) according to the manufacturer’s instructions. Ten μg of *B. anthracis* RNA was enriched twice, resulting in high quality mRNA with minimal ribosomal RNA contamination. Double-stranded cDNA templates were generated from 500 ng of bacterial RNA using selective non-ribosomal primers (Ovation® Prokaryotic RNA-Seq System, NuGen Technologies, San Carlos, CA) and Superscript III reverse transcriptase (Life Technologies) as previously described [31].

### RNA-seq library preparation

The TruSeq DNA Sample Preparation Kit (Illumina, San Diego, CA) and its workflow were used to complete the RNA-seq library preparation, using the synthesized cDNA as template, with some modifications. Purification of the ligated products was performed using a Pippin Prep DNA size selection system (Sage Science, Beverly, MA). The 1.5% (w/v) agarose gel cassette was used for the isolation of library fragments in the 400 to 600 bp range. Size-selected products were purified using a 1:1 ratio of Agencourt AMPure XP beads (Beckman Coulter Genomics, Danvers, MA) to product, and eluted in 20 μL EB Buffer (Qiagen). The second modification was to increase the number of amplification cycles to 14 in order to keep the workflow consistent with Illumina’s TruSeq RNA Sample Preparation Guide. The final library products were quantified by qPCR using a KAPA Illumina GA Library Quantification Kit (KAPA Biosystems, Woburn, MA) and sequenced on a Hiseq 2000 (Illumina).

### Read mapping

The 50-bp Illumina HiSeq reads were first trimmed of adapter sequence using custom software tools and further trimmed and filtered for low quality sequence using the FASTX toolkit (Hannon Lab, Cold Spring Harbor Laboratories, Cold Spring Harbor, NY). Processed reads were mapped to the *B. anthracis* genome using ZOOM (Bioinformatics Solution Inc., Waterloo, Canada). Uniquely mapped reads were analyzed to determine the read counts per protein-coding gene.

### Identification of genes with differential expression between conditions

We define a genotype as the presence or absence of the gene AtxA, and an environment as the presence or absence of added CO_2_ in the medium. Based on these factors, we defined four conditions on the basis of their possible combinations: (1) Ames 35 wildtype strain grown in NBY and air (WT/air), (2) Ames 35 wildtype strain grown in NBY in the presence of CO_2_ (WT/CO_2_), (3) AtxA deletion strain grown in NBY and air (ΔAtxA/air), and (4) AtxA deletion strain grown in NBY and CO_2_ (ΔAtxA/CO_2_). The two biological replicates per condition allowed us to attempt to distinguish whether differences observed in expression were due to random fluctuations between replicates or to true regulatory changes. We then calculated fold-changes between conditions using normalized count values for each protein-coding gene and we eliminated technical bias using the reads per kilobase per million base pair exon model (RPKM) statistic [32]. Furthermore, for each gene, the expression counts across all 8 samples were summed and the sum was compared between all genes. Genes in the bottom 30^th^ percentile were filtered out [33]. One challenge in the analysis of high-throughput sequencing data is the determination of significant differences between samples and conditions*, i.e*. differences that are significantly higher than the random variation between samples. Because of the controversy of using the Poisson distribution, which in some cases may be too restrictive [34], we used the DESeqR package that is based on negative binomial distributions [34]. Using this approach, we fit four negative binomial generalized linear models for each gene:

(1) ReadCounts∼Genotype

(2) ReadCounts∼Environment

(3) ReadCounts∼Genotype+Environment

(4) $$\begin{array}{l} \mathrm{Read}\;\mathrm{Counts}∼\mathrm{Genotype}+\mathrm{Environment}\\ \;+\mathrm{Genotype}:\mathrm{Environment} \end{array}$$

To test whether each factor has a significant impact on the expression of a given gene, the goodness of fit between the models were compared with likelihood-ratio χ^2^ tests. The results from comparing the fits of (3) to (1) and (3) to (2) are called the main effects of environment and genotype, respectively. For significance, the p-value cut-off for the main effects was set to 0.01 and we required a fold-change difference of at least 2 between conditions (i.e., WT and ΔAtxA or CO_2_ and air). The results from comparing the fits of (4) to (3) are called the interaction effects, and results were called significant when p-values were less than 0.01 and when there was a fold-change difference of at least 4 across genotype-environment pairings (i.e., between WT/CO_2_ and ΔAtxA/air, or between WT/air and ΔAtxA/CO_2_). The p-value was chosen to be relatively stringent to help account for the relatively small number of biological replicates per condition. All p-values were corrected for multiple comparisons by maintaining the genome-wide false discovery rate [35]. Since expression counts may vary based on plasmid copy number, we performed these analyses separately for the chromosome and for pXO1.

Since we refer to it often, we call the set of genes with a significant interaction effect and increased expression in the two WT/CO2 samples compared to the two ΔAtxA/air samples the “virulence condition synergy group” (VCSG). We conducted these analyses using the R programming language and processed the results into tables using Excel 2010 (Microsoft, Redmont, WA). The script is available at https://github.com/andymckenzie/AtxA.

### Bioinformatic analysis of clusters of genes upregulated in virulence conditions

We used BLAST to find homologous genes [36] and ClustalX to align homologous nucleotide sequences [37]. To search for conserved protein domains, NCBI’s Conserved Domain Database was used [38]. Additionally, we searched for the presence of gene operons in the virulence condition synergy group. Two adjacent genes were defined as in an operon if they had fully contiguous (i.e., non-zero) read mapping from the translation start site of the upstream gene to the translation stop site of the downstream gene in both WT/CO_2_ RNA samples [28]. Pseudogenes, defined by the NCBI annotations as non-functional on the basis of evolutionary comparisons, were not included in operons.

### Bioinformatic analysis of potential small RNAs

MochiView was used as a genome browser to visualize levels of RNA expression across the chromosome and pXO1 [39]. Through visual inspection, regions of high expression were detected which did not map to any protein-coding regions, which was suggestive of the presence of small, non-coding, regulatory RNAs (sRNAs). The transcript boundaries of the sRNAs were treated as the equivalent translation start and stop sites for read mapping and subsequent differential expression analysis. To evaluate these putative sRNAs, minimum free energy RNA secondary structure predictions were made using RNAfold [40]. On RNAfold, all of the default parameters were used, including the option to avoid isolated base pairs. RNApredator [41] was used to search for sRNA binding sites. Because of the difficulty of computational searches for sRNA binding sites [42], we restricted this search to pXO1 (NC_007322), which is where the putative sRNA genes were located. For predicted interaction sites located upstream of translation start codons, we determined whether that site was expressed contiguously with the rest of the transcript in both WT/CO_2_ samples. If not, the prediction was discarded. In the case of duplicates, the prediction as belonging to the longer ORF was reported.

### qPCR validation of RNA-sequencing results

Seven genes, including the two sRNAs, were selected for qPCR validation of the RNA-seq data. Three constitutively expressed reference gene candidates were selected from the RNA-seq data based upon coefficient of variation (CV) and their expression level. All primers and fluorescent probes (Additional file 1: Table S2) were designed using Primer Express version 3 software (Life Technologies) and purchased from Biosearch Technologies (Novato, CA). Each of the seven candidate gene primers and probes were tested for PCR amplification efficiency in multiplex with three candidate reference genes using absolute qPCR method (Life Technologies, Carlsbad, CA). QPCR reactions were carried out in triplicate in 20 μL containing 5 μL template DNA, 1X Express qPCR Supermix with premixed with 5-carboxy-X-rhodamine, triethylammonium salt (ROX; Life Technologies, Carlsbad, CA), two sets of 400 nM forward and reverse primers, and 120 nM fluorescent probe both for reference gene and the candidate gene. Q-PCR reactions were carried out at 50°C for 2 min, 95°C for 2 min, and 55 cycles of 95°C for 15 sec and 60°C for 1 min. Data were analyzed using 7900HT version 2.3 sequence detection system software according to the manufacturer’s recommendations (Life Technologies, Carlsbad, CA). PCR amplification efficiency (E = (10^(-1/slope)^-1) of each reference gene in combination with each target gene (Additional file 1: Table S2) were calculated. PCR amplification efficiency of the reference gene aspartate kinase (dapG-2) in combination with 7 gene candidates was the best of the three reference genes tested. All multiplex PCR reactions with dapG-2 gene primers and probe had PCR amplification efficiency greater than 94%. Prior to synthesis of Q-PCR template cDNA, RNAs were tested for contaminating genomic DNA. DapG2 gene primers and probe (Additional file 1: Table S2) were used to quantitate genomic DNA using reference *B. anthracis* DNA using the absolute quantitation method (Life Technologies, Carlsbad, CA). Genomic DNA contamination was less than 0.005% of the total nucleic acid in all eight samples. cDNAs were made from 500 ng of bacterial RNA using SuperScript VILO cDNA synthesis kit according to the manufacturer’s protocol (Life Technologies, Carlsbad, CA). cDNAs were purified using acid phenol:chloroform:isoamylalcohol (Life Technologies, Carlsbad, CA) as described before [31]. Each mRNA/sRNA copy number was normalized to dapG-2 mRNA copy number according to the comparative quantitation method as described before [31]. Pearson’s correlation was calculated between the RNA-sequencing read count and normalized qPCR values using the Partek Genomics Suite (Partek, St. Louis, MO).

## Results

### Generation and complementation of an AtxA deletion mutant

**Figure 1:**
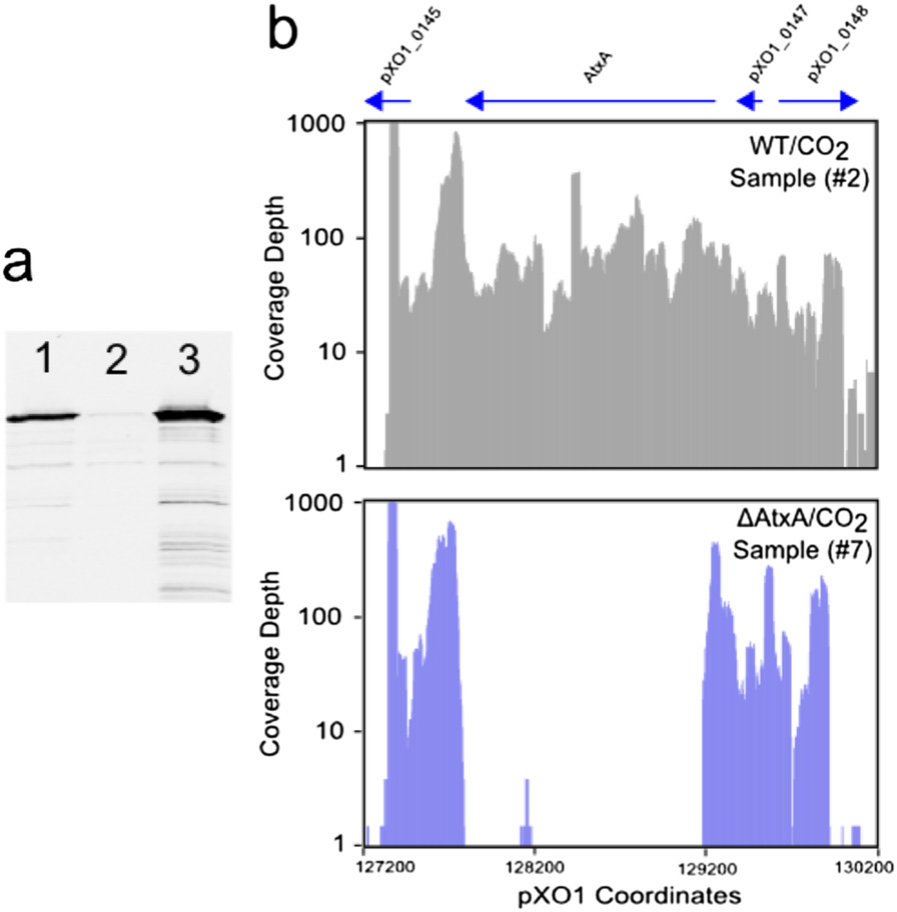
**Complementation of ΔAtxA mutant and visualization of expression differences.** Western blot expression analysis of protective antigen is shown in **(a)**. Lane 1, Ames 35. Lane 2, Ames 35 ΔAtxA. Lane 3, Ames 35 ΔAtxA + pIU68. **(b)** MochiView images of read counts for the area of pXO1 overlapping the AtxA ORF in representative WT and ΔAtxA samples. The y-axis is normalized to the highest count value in the region and transformed to a log base-2 scale.

This work was designed to identify *B. anthracis* genes that respond to CO_2_ through mechanisms involving the key virulence gene regulator AtxA. To do this in a well-defined way, a knockout of *atxA* was created and subsequently complemented. We applied the current version of the Cre-LoxP system that was developed in our laboratory [29] to substitute the *atxA* gene with a single loxP site, as outlined in the Methods section, and we confirmed the genotype by PCR and DNA sequencing. Since laboratory culture of *B. anthracis* can lead to undesired, second-site mutants [43], we performed *atxA* complementation controls by expressing AtxA from plasmid pIU68 [14]. Following growth to similar OD600 values we visualized the relative amounts of PA secreted by the strains and isogenic mutants by Western blotting of concentrated culture supernatants. PA expression was observed to be absent in the AtxA deletion strain, as was expected, whereas the parental phenotype was restored by the addition of pIU68, leading to PA production at a level somewhat exceeding that in the parental Ames 35 strain (Figure 1(a)). The higher expression in the complemented strain may be due to production of AtxA by the multicopy plasmid pIU68 at concentrations exceeding that in the WT strain.

### Transcriptome analyses

Global transcriptional analyses were performed on the parent Ames 35 strain and the AtxA deletion mutant under growth conditions described above. Briefly, RNA samples that were prepared from mid- to late-log phase cultures were converted to cDNA and sequenced on the Illumina platform. Data were obtained from duplicate samples of four conditions, differing in genotype (WT vs. AtxA mutant) and growth environment (ambient air vs. CO_2_). The eight samples behaved similarly (Table 1), with the exception of sample #5, which produced fewer reads, while all other samples produced at least 2 × 10^7^ unique reads.

**Table 1:** Summary of RNA-sequencing results

**Sample ID**	**Condition**	**OD600**	**Total reads**	**Unique reads**	**Unique reads (%)**	**Gene reads**	**Gene reads (%)**
1	WT/CO_2_	2.0	62,677,017	35,654,298	57	29,959,488	84
2	WT/CO_2_	2.1	63,937,489	40,947,059	64	34,294,534	84
3	ΔAtxA/air	2.0	69,132,947	32,303,226	47	27,284,918	84
4	ΔAtxA/air	1.9	67,144,286	34,755,237	52	30,494,637	88
5	WT/air	2.0	68,812,647	23,082,971	34	17,928,149	78
6	WT/air	2.2	63,046,363	45,881,828	73	39,525,419	86
7	ΔAtxA/CO_2_	2.0	60,638,322	39,860,432	66	34,405,268	86
8	ΔAtxA/CO_2_	2.3	66,526,879	34,284,152	52	28,722,268	84

OD600 indicates the optical density at which each bacterial sample was subjected to RNA isolation. Total reads represent the number of high-quality reads. Unique reads represents reads that mapped to unique portions of the *B. anthracis* genome or pXO1 plasmid, excluding ribosomal RNA. Unique reads (%) was calculated as the ratio of unique reads to total reads. Gene reads refers to the number of unique reads that mapped to known protein-coding genes. Gene reads (%) was calculated as the ratio of gene reads to unique reads.

An immediate positive control in our analysis was confirmation of the *atxA* deletion in the constructed mutant (Figure 1(b)). In the WT strain (Samples 1, 2, Table 1) the number of reads mapping to the *atxA* gene were similar to those of adjacent genes. Of note, there were only a few reads within the *atxA* gene in the AtxA-deficient strain, reaching no more than a coverage depth of 10 (Samples 7,8, Table 1). The same patterns were seen in all eight samples. Expression patterns of genes adjacent to *atxA*, however, appeared very similar, indicating that the deletion had no effect on the transcription of neighboring genes. Of note, in our wildtype samples, *atxA* had a non-significant fold-change increase of 1.15 between CO_2_ and air environments.

### Expression patterns of genes upregulated in virulence conditions

We grouped *B. anthracis* genes into gene-sets on the basis of their differential expression in virulence-related conditions. AtxA primarily increases the expression of the genes it regulates, while acting synergistically with environments containing CO_2_[11]. Thus, we were particularly interested in genes whose expression patterns displayed a greater than additive interaction in the presence of both AtxA and CO_2_. Using a likelihood ratio test of the interaction between genotype and environment and a fold-change cut-off between WT/CO_2_ and ΔAtxA/air conditions, we identified genes that were upregulated in a synergistic manner in the presence of both AtxA and CO_2_. This set of genes, which we designate here as the Virulence Condition Synergy Group (VCSG), contains fifteen genes encoded on pXO1 and three genes encoded on the chromosome (Table 2). Interestingly, the pXO1-specific gene set included two small RNAs (sRNAs) demonstrating properties of small, non-coding, regulatory RNAs. For purposes of discussion, these two sRNAs are designated as sRNA-1 and sRNA-2, and they reside from 105,771 to 105,909 and 131,397 to 131,525, respectively, on the pXO1 plasmid. These putative sRNAs will be described in more detail in a later section in this manuscript. For completeness, we also include tests investigating the genes significantly changed in all other comparisons, which are listed in Additional file 1: Tables S3-S9. Of the other interaction effects, we identified 17 genes with increased expression in conditions of the ΔAtxA genotype and air environment, 1 gene with increased expression in conditions of the ΔAtxA genotype and CO2 environment, and 23 genes with increased expression in conditions of the wildtype genotype and air environment. We did not perform systemic gene function analyses on the differentially expressed sets of genes, as is often the custom for microarray and RNA-seq analyses, because the number of genes that are significantly differentially expressed between the WT and AtxA knockout strains is relatively small, and gene function enrichment tests rely on relatively larger sample sizes in order to make robust inferences.

**Table 2:** Genes of the virulence condition synergy group

**Locus tag**	**Gene name**	**Accession number (NCBI)**	**Adjusted P-Value**	**Fold-change**	**Length of predicted protein (AA)**	**Putative function**
pXO1_0122		YP_016453.2	0.0074	5.0	158	Hypothetical protein
	*sRNA-1*		< 0.0001	338.5		Unknown
pXO1_0123		YP_016454.2	0.0008	68.2	34	Hypothetical protein
pXO1_0124	*bslA*	YP_016455.2	< 0.0001	192.1	652	Adhesin; virulence factor
pXO1_0125		YP_016456.2	< 0.0001	42.1	280	PAP2 family phosphatase
pXO1_0137		YP_016468.2	0.0008	63.7	151	Hypothetical protein
pXO1_0142	*cya*	YP_016473.2	0.0065	4.1	800	Edema toxin; virulence factor
	*sRNA-2*		< 0.0001	332.1		Unknown
pXO1_0151		YP_016482.2	< 0.0001	141.8	44	Hypothetical protein
pXO1_0153		YP_016484.2	< 0.0001	69.1	76	Hypothetical protein
pXO1_0164	*pagA*	YP_016495.2	< 0.0001	130.9	764	Protective antigen; virulence factor
pXO1_0165		YP_016496.2	< 0.0001	84.2	34	Hypothetical protein
pXO1_0166	*pagR*	YP_016497.2	< 0.0001	126.2	99	Transcriptional regulator
pXO1_0171		YP_016502.2	< 0.0001	17.7	68	Hypothetical protein
pXO1_0172	*lef*	YP_016503.2	0.0024	30.9	809	Lethal toxin; virulence factor
GBAA0793		YP_017427.1	0.0002	10.9	436	Phosphotransferase system
GBAA0795		YP_052611.1	<0.0001	9.7	361	DUF871 superfamily
GBAA2301		YP_018949.1	<0.0001	7.5	90	YokU superfamily

This table lists genes whose expression patterns are best explained by a synergistic relationship between the presence of AtxA and CO_2_. P-values were determined using a likelihood ratio test of the interaction between genotype and environment. Fold-change refers to the ratio of the counts mapping to that gene in conditions of WT/CO_2_ over ΔAtxA/air. Gene functions listed are those determined by validated experiments in *B. anthracis* or the primary conserved domain is indicated if validated data are lacking. In the absence of conserved domains, the function is listed as a “Hypothetical protein”.

**Figure 2:**
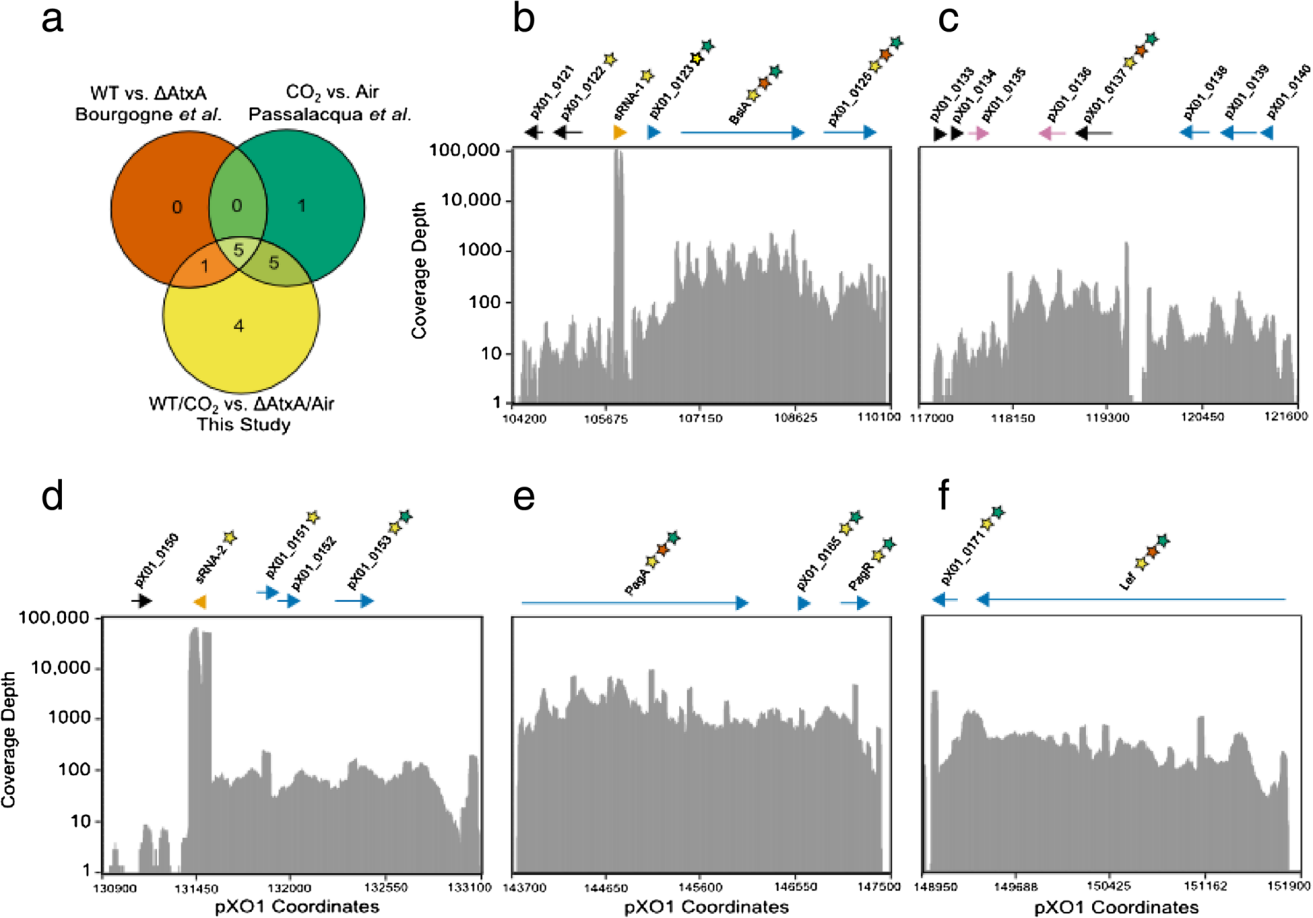
**Regions containing genes in virulence condition synergy group and comparisons to previous data. (a)** Venn diagram showing the overlap between the number of genes on pXO1 whose expression were significantly upregulated in two previous studies [12,27] vs. those from the current study. **(b-f)** Plots displaying representative count data of one of the two WT/CO_2_ sample (Sample 2, Table 1) along indicated regions of pXO1 transformed to a log base-2 scale. The y-axis is normalized to the highest expression on pXO1 and remains the same for all five plots. Colors indicate the class of gene: blue for protein-coding genes considered to be in operons, purple for pseudogenes, orange for sRNAs, and black for the remaining protein-coding genes. The longest open reading frame (for protein-coding genes) or transcript boundaries (for sRNAs) are mapped above the plots. Stars mark the genes whose expression were upregulated in Bourgogne *et al.* (red), Passalacqua *et al.* (green), and this study (yellow). Note that these five plots are not an exhaustive representation of all genes upregulated on pXO1 and so do not enumerate all genes counted in (A).

We compared the list of genes identified as members of the VCSG to lists generated in two previous reports that were based on microarray data. The first of these studies [27] identified six genes that were upregulated in a WT strain compared to an AtxA-deficient strain. The second study [12] identified 11 genes that were upregulated in a WT strain grown under CO_2_-containing conditions relative to ambient air. Although each of these analyses employed different statistical criteria and technologies, the results for genes encoded on pXO1 were similar to those found in our study (Figure 2(a)), with some differences. For example, our study identified 15 genes differentially regulated by either AtxA deficiency or increase in CO_2_ concentration or the synergistic action of both. All genes on pXO1 that were found in the two previous studies to be regulated by either CO_2_ or separately by AtxA, were also found in this study, and they were found to be regulated by the interaction of CO_2_ and AtxA. In contrast, of the chromosomally-encoded genes, only one of the three genes described here, for which we detected a significant interaction between CO_2_ and AtxA (GBAA2301), was also found by Passalacqua *et al*. to be greater than 2-fold upregulated in CO_2_ compared to air [12].

### Putative operon organization of genes

We observed that genes of the VCSG are often clustered, suggesting their possible organization into operons for coordinated expression and regulation. For example, genes *pXO1_0123* through *pXO1_0125* are contiguous (Figure 2(b) and their relatively similar levels of expression suggest that they may constitute an operon. Adjacent and upstream of this putative operon is the small RNA sRNA-1, which was expressed at a very high level as compared to *pXO1_0123* through *pXO1_0125.* For the three genes *pXO1_0123* through *pXO1_0125*, more reads mapped to the region representing the central *bslA* gene (*pXO1_0124)* (Figure 2(b)) than to the flanking genes. This may be due to mRNA stability being greater within the *bslA* coding region rather than at the 3′ or 5′ ends of the polycistronic mRNA. Alternatively, promoter elements specific to *bslA* expression may be present (Figure 2(b)). First, these promoter elements could be present within the coding regions of the upstream genes, and second, the intergenic distances between sRNA-1, *pXO1_0123*, *pXO1_0124*, and *pXO1_0125* are 441, 369, and 270 base pairs, respectively, which also provide room for potential promoters. Of note, none of these intergenic regions lacked reads, as would be the case if any transcripts terminated between the *pXO1_0123*, *pXO1_0124*, and *pXO1_0125* genes. BslA mediates adhesion to host cells, and has been found to mediate the ability of the bacterium to cross the host blood-brain barrier. Because BslA is an established virulence factor [44-46], it is not surprising that *bslA* transcripts are abundant relative to the flanking genes. The evidence that expression of *pXO1_0123* and *pXO1_0125* is coordinated with that of *bslA* suggests that they serve important functions, a suggestion that merits further study.

We identified another region on pXO1 that contains several clusters of upregulated genes and these data are shown in Figure 2(c). Expression of the genes *pXO1_0138*, *pXO1_0139*, and *pXO1_0140*, which are coded in the same (3′ to 5′) orientation, was increased by greater than 2-fold in the WT/CO_2_ samples compared to ΔAtxA/air. This result is consistent with data reported in one of the earlier microarray studies [12]. Currently, no known function or annotation exists for these three open reading frames. The intergenic distances between genes *pXO1_0138*, *pXO1_0139*, and *pXO1_0140* are 130 and 54 base pairs, respectively. Given these small intergenic distances and the overlapping and near-equal number of reads for these genes, they appear to constitute an operon. Downstream of *pXO1_0138* is a gap where the lack of reads is evident, suggesting termination of the mRNA for genes *pXO1_0138*, *pXO1_0139*, and *pXO1_0140* at this location. On the left side of Figure 2(c) reside two additional clusters of genes that we found to be upregulated in WT/CO_2_. These clusters of genes consist of *pXO1_0133* through *pXO1_0135* and of genes *pXO1_0136* and *pXO1*_0137. The intergenic distances for genes *pXO1_0133* through *pXO1_0135* are 86 and 31 base pairs, respectively. While genes *pXO1_0135* and *pXO1_0136* are annotated as pseudogenes as a result of frameshift mutations, our data show they are highly expressed. All these transcripts were upregulated in WT/CO2, but only *pXO1_0137* met all the criteria for inclusion in the VCSG (Table 2).

Indicative of another CO_2_-responsive, clustered gene set on pXO1 is the contiguous high expression of *sRNA-2* and of the *pXO1_0151*, *pXO1_1052*, and *pXO1_0153* genes (Figure 2(d)). Of interest, sRNA-2 is located upstream and transcribed in the opposite direction from the three other genes, so it cannot be considered part of their operon. The three genes are predicted to encode very short proteins, ranging from 45 to 77 amino acids, of unknown function. Only three of these genes qualify for inclusion in the VCSG, with *pXO1_0152* not qualifying simply because of its very short putative coding region, even though its transcript level is obviously equal to that of the flanking genes.

Of particular interest to us was the region on pXO1 that includes the anthrax protective antigen gene *pag A (pXO1_0164)* and its repressor *pagR (pXO1_0166),* together with an intervening gene *(pXO1_0165)* that has no known function (Figure 2(e)). Promoter regions upstream of the *pagA* start codon containing as little as 150 bp are sufficient to yield an increase in expression in environments containing CO_2_[47,48]. Two transcripts have been described for this operon, a 4.2-kb transcript corresponding to the entire region, and a truncated 2.7-kb transcript resulting from the action of an attenuated transcription terminator downstream of *pagA*[49]. Our data demonstrate that for the conditions studied here, there was only a slight decrease in transcript amounts at the distal end containing *pagR* (Figure 2(e)). Thus, our data indicate that differential expression between *pagA* and *pagR* is not pronounced, at least in the conditions used here.

While the gene encoding edema factor *(cya, pXO1_0142)* was upregulated in a monocistronic manner (data not shown), the gene encoding lethal factor (*lef, pXO1_0172*), appears to be part of an operon that includes the downstream gene *pXO1_0171* (Figure 2(f)). This gene encodes a protein with no known function. Expression for *pXO1_0171* is lower than seen for *lef*, which may suggest 3′ degradation of the polycistronic message. Taken together, the RNA-seq data described above provide a new level of detail regarding coordinated expression of gene sets on pXO1 relative to what has previously been reported. The many genes found here to be upregulated under virulence-inducing conditions, but having no known function, deserve additional study.

Although we point out above that many of the VCSG genes in the pathogenicity island are in operons, we do not suggest that operonic organization of genes is enriched within the pathogenicity island versus other regions. There is little reason to suggest such anenrichment, and the number of genes is too small to warrant a statistical analysis of their distribution.

### Small RNAs with high expression in WT/CO_2_ conditions

**Figure 3:**
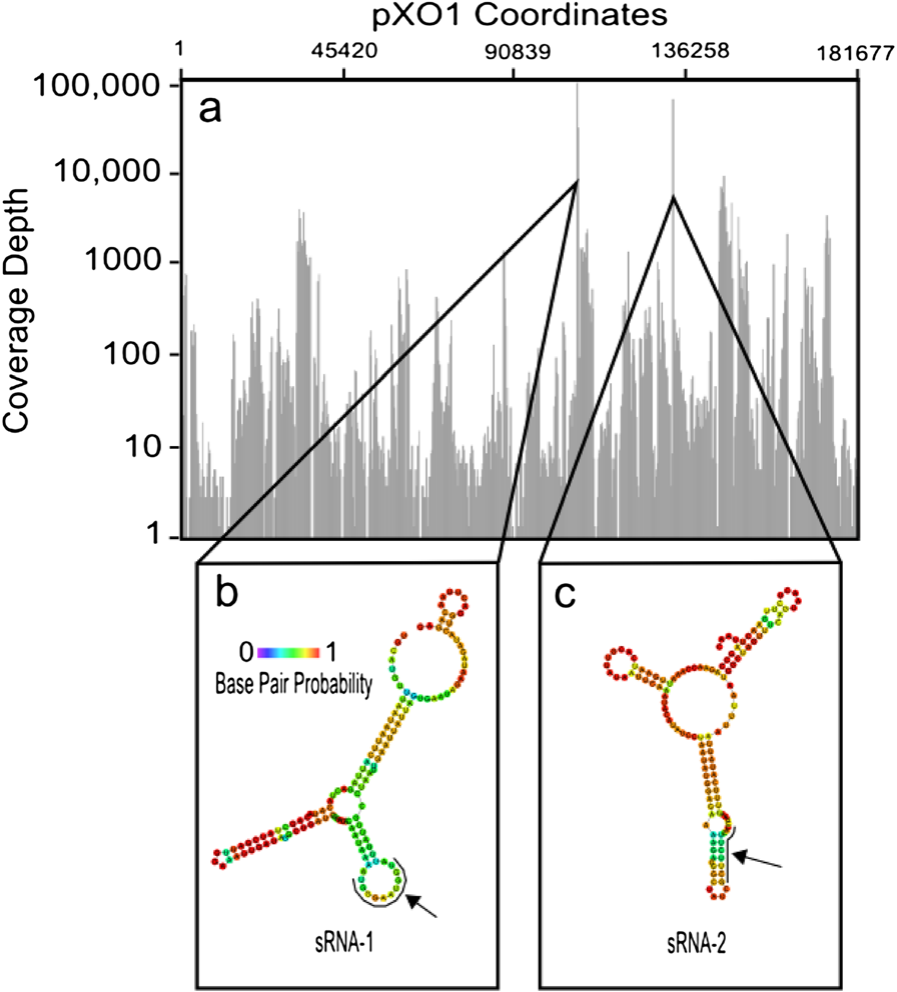
**Genomic locations, expression levels, and secondary structures of highly expressed sRNAs. (a)** Representative WT/CO_2_ sample (Sample 2, Table 1) showing the distribution of read maps on pXO1. The y-axis is normalized to the highest count value in the region and transformed to a log base-2 scale. **(b, c)** RNAfold-generated minimum free energy structure predictions of the two sRNAs. Arrows indicate the 10-bp intervals most commonly found in the most likely sRNA-mRNA binding predictions, which are coordinates 75-84 for sRNA-1 and 68-77 for sRNA-2.

As noted above, our transcriptome analysis revealed two small RNAs, *sRNA-1* and *sRNA-2*, encoded on pXO1, that were highly expressed in WT/CO_2_. With predicted transcript lengths of 139 and 129 base pairs, respectively, these small RNAs fit the definition of bacterial regulatory sRNAs [50]. A pXO1-wide display of WT/CO_2_ sequence read numbers (Figure 3(a)) showed these transcripts to be at least 10-fold more abundant than all other transcripts measured. Since our RNA sequencing data are non-directional, we proceeded to analyze an independent tiling microarray dataset of Ames 35 grown in a CO_2_-containing environment to determine the transcriptional direction of the sRNAs (Hu *et al*., unpublished data). This analysis showed that sRNA-1 is transcribed on the coding strand while sRNA-2 is transcribed on the complementary strand (Figure 2(b) and (d)). We then analyzed the sequences of three pXO1-like plasmids, pCI-XO1 [51], pBCXO1 [52], and p03BB102_179 [53] and discovered that sRNA-1 is 100% identical to a region in each of these plasmids. The other small RNA, sRNA-2, is 99% homologous to corresponding putative sRNAs encoded on the same plasmids. Thus, both sRNAs that we have discovered appear to be highly conserved in other pXO1-like plasmids, suggesting they may have conserved functions across strains and plasmids of other *B. anthracis* strains and isolates.

The most common function of regulatory sRNAs is to bind to complementary mRNAs and increase, or, more commonly, decrease mRNA translation [42]. We used RNApredator, a sRNA-mRNA binding prediction tool, to identify putative targets for sRNA-1 and sRNA-2 on pXO1, and we report here the five most probable targets (Table 3). sRNA-1 was predicted to bind to the 5′-untranslated region (UTR) of the transcript of *atxA*, starting at 129 bases upstream from the start codon, via a perfect complementary sequence of the 10-bp region seen in Figure 3(a). For both sRNAs, the most likely sRNA-mRNA binding regions are indicated in Figure 3(b) and (c). Another potential target of sRNA-1 was discovered in *pXO1_0199* (Table 3), starting at 184 bases downstream from the start codon. *pXO1_0199* is annotated as related to *relA*, which is known to play an important role in stress responses [54]. sRNA-2 was predicted to bind to transcripts of a variety of genes on pXO1. Of note, these genes encode proteins that to date have not been associated with virulence.

**Table 3:** Predicted mRNA binding targets of sRNA-1 and sRNA-2 on pXO1

**Small RNA**	**Closest ORF (Locus Tag)**	**Protein name or ID**	**Region of predicted mRNA**	**Region of redicted sRNA**	**Energy of interaction (kJ/mol)**	**Z-score**
sRNA-1	pXO1_0199	relA-like	184-196	125-137	−13.98	−2.13
	pXO1_0146	AtxA	(-138)-(-129)	75-84	−13.53	−2.01
	pXO1_0113	YP_016444.2	239-259	62-82	−12.79	−1.82
	pXO1_0080	S-layer domain protein	637-653	116-137	−12.47	−1.73
	pXO1_0182	Integrase/recombinase	20-29	75-84	−12.05	−1.62
sRNA-2	pXO1_0066	YP_016397.2	1079-1103	61-86	−14.47	−3.21
	pXO1_0067	RepX (FtsZ-like)	268-278	68-78	−13.36	−2.72
	pXO1_0010	YP_016341.2	178-199	61-78	−13.32	−2.7
	pXO1_0130	UDP-glucose 6-dehydrogenase	1006-1023	69-86	−12.53	−2.35
	pXO1_0113	YP_016444.2	107-132	77-104	−12.52	−2.35

RNAPredator predictions of the five mRNA targets having the lowest Z-scores for each sRNA are listed. Z-scores quantify the deviation of the binding energy for that interaction from the set of all possible sRNA-mRNA binding interactions.

### Correlation of RNA-sequencing expression data to qPCR

To validate our RNA-seq data, we measured the expression of seven genes, including *sRNA-1* and *sRNA-2*, by qPCR analysis. For all seven genes, the Pearson correlation coefficients between the RNA expression values and qPCR quantitations were greater than 0.85 (Table 4, Additional file 1: Figure S2). Therefore, the RNA results we discuss here are supported by two different technologies with high correlation.

**Table 4:** Correlation of qPCR expression with RNA-sequencing expression for selected genes

**Locus tag**	**Name and/or function of product**	**Pearson’s R**	**P-Value**
sRNA-1	Unknown	0.99	< 0.0001
GBAA_pXO1_0124	bslA, adhesin	0.99	< 0.0001
sRNA-2	Unknown	0.99	< 0.0001
GBAA0887	Eag, surface layer protein	0.89	0.0077
GBAA1639	GerN, germination protein	0.99	< 0.0001
GBAA2840	Transporter, putative	0.99	< 0.0001
GBAA4875	Universal stress protein family	0.86	0.0127

For qPCR, gene expression was normalized to the expression of the housekeeping gene, GBAA3936, encoding aspartokinase. Sample Pearson correlation coefficients were calculated for each gene in all eight samples.

### Position of genes upregulated in virulence conditions along pXO1 and the chromosome

**Figure 4:**
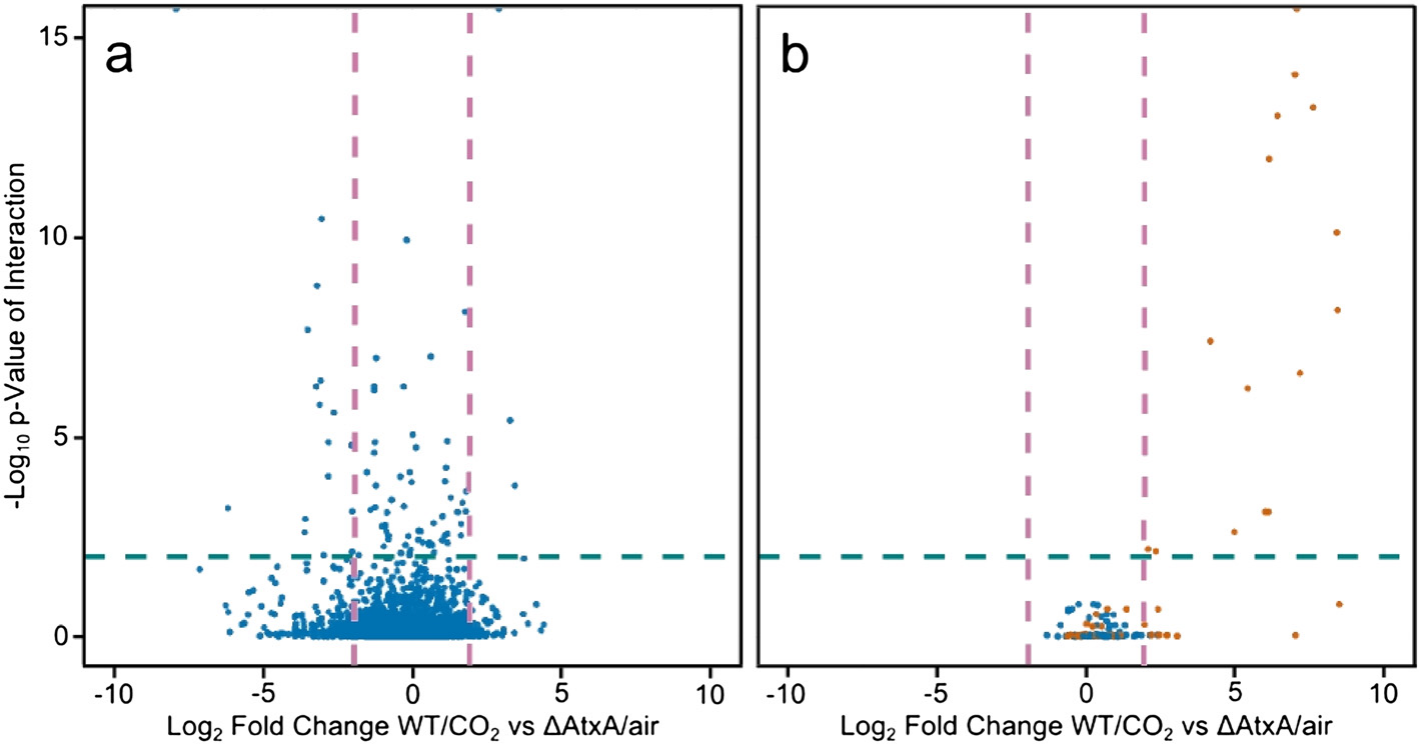
**Distribution of expression changes in WT/CO2 samples and p-values of the interaction effect.** Scatter plots showing the joint distribution of fold-change between WT/CO_2_ and ΔAtxA/air (transformed to a log base-2 scale) and p-values of the likelihood ratio test for the genotype by environment interaction (transformed to a negative log base-10 scale) on the chromosome **(a)** and pXO1 **(b)**. Only genes above the 30^th^ percentile of total expression are included. The teal dotted line shows the adjusted p-value cutoff at 0.01. For pXO1, genes in the 58.9-kb extended pathogenicity island are colored in orange.

To graphically represent the frequency and location (genome vs. plasmid) of genes that respond to CO_2_ and AtxA, we generated modified volcano plots, which are scatter plots often used in gene expression studies [55] (Figure 4). These plots compare the p-values for significant interaction effects with the fold-expression differences between the conditions of WT/CO_2_ and ΔAtxA/air. Most of the chromosomally-located genes whose expression showed a significant interaction effect between genotype and environment did not show large fold-change differences between these two conditions (Figure 4(a)). In contrast, all of the genes on pXO1 with a significant interaction effect also have a greater than 4-fold increased expression in WT/CO_2_ vs. ΔAtxA/air samples (Figure 4(b)). Many of these genes on pXO1 are found in a region previously defined as a pathogenicity island, and having boundaries defined by inverted *IS1627* elements located at coordinates 117,177 and 162,013 of pXO1 of the Sterne strain [1]. The corresponding boundaries of the pathogenicity island for the pXO1 plasmid from Ames 35 (used in this study) are at coordinates 116,085 and 163,674 (NC_007322.2). This region is close to, but does not include, the small RNA *sRNA-1* or *bslA*. Thus, we suggest extending the left-side boundary of the pathogenicity island to a point 1000 bp upstream of the start of the *sRNA-1* transcript (to base coordinate 104,771) while retaining the right-side boundary of the traditional pathogenicity island, coordinate 163,674 for the pXO1 plasmid from Ames 35. In this study, all genes on pXO1 that were significantly upregulated in the WT/CO_2_ VCSG map to this expanded 58.9-kb pathogenicity island. Their expression levels are highlighted in red in Figure 4(b). The ratio of the number of genes in the VCSG on the extended pathogenicity island to the number of bp in the extended pathogenicity island is 3.08-fold higher than the ratio calculated with number of bp in pXO1 as a whole as the denominator. Further, the ratio of the number of genes in the VCSG on the extended pathogenicity island to the number of bp in the extended pathogenicity island is 444-fold higher than the ratio of the number of genes in the VCSG on the chromosome to the number of bp on the chromosome.

## Discussion

In this study, we searched for genes whose expression is altered by the presence of two factors known to affect the production of virulence factors: CO_2_ and the transcriptional regulator AtxA. Genotype-environment interactions occur when the effect on expression of the genotype and environment are not independent, which is a common finding in gene expression studies [56]. We were particularly interested in genes which show a synergistic dependence on AtxA and CO_2_. Using an RNA-seq approach, we identified 15 genes on the *B. anthracis* virulence plasmid pXO1 and three genes on the chromosome that are significantly upregulated in expression due to the combined presence of AtxA and CO_2_. One limitation of our analysis is that we have only two biological replicates per condition, but we address this limitation in part by requiring relatively strict p-values and fold-change values in order for a gene to be considered significantly differentially expressed. Of particular interest, we discovered two novel sRNAs encoded on pXO1 whose expression is greatly increased in the presence of both CO_2_ and AtxA. Many of the remaining CO_2_- and/or AtxA-responsive genes on pXO1 had been identified in previous studies, so our data support their identification and the characterization of their operon-like functions. Equally important is our observation that the majority of genes that are upregulated separately by CO_2_ or AtxA are also upregulated synergistically by their combination. Understanding the interaction of these key components is necessary for a full description of how ancillary genes are transcribed in association with the known pXO1-encoded toxins. Furthermore, our findings add to the number of pXO1-encoded genes that are linked to and affected by AtxA and CO_2_. These genes offer subjects for future experiments aimed at characterizing the genetic systems involved in the pathogenesis of *B. anthracis*.

Earlier studies reported data consistent with the presence of the two sRNAs that we defined and discussed in detail here. One study used microarrays to analyze the effects of deleting the quorum-sensing *luxS* gene on the transcriptome of *B. anthracis* grown in medium with 0.8% NaHCO_3_[57]. This study noted a region of high transcription near the location for *sRNA-1*. However, lack of annotation and therefore array probe coverage of this region apparently prevented recognition that this was a small RNA. An independent RNA-sequencing study of *B. anthracis* grown under CO_2_-containing conditions [28] also identified high gene expression in the regions near the two sRNAs we describe here. Examination of the primary data deposited by the authors showed that more reads mapped to the transcript boundaries of *sRNA-1* and *sRNA-2* than to any other region of pXO1. However, the authors’ publication did not discuss the presence of these transcripts. Thus, our data both confirm and extend results obtained in the previous studies.

Since both *sRNA-1* and *sRNA-2* are highly upregulated in conditions containing both AtxA and CO_2_, we hypothesize that these molecules may play a role in regulating virulence gene expression pathways or other gene targets or pathways. One candidate target of sRNA-1 is *atxA* (Table 3), which is thought to be regulated post-transcriptionally by an unknown mechanism [24]. Interestingly, we discovered that *atxA* contains a perfect 10-bp region complementarity to sRNA-1 within its 5’ UTR. While this suggests that sRNA-1 might regulate the transcription of atxA, existing data and our own unpublished data show that the levels of AtxA vary by at most 2-fold in response to environmental conditions. In contrast to the potential genetic targets of sRNA-1, sRNA-2 was not predicted to target any of the known virulence factors on pXO1. This result suggests that sRNA-1 may have an *atxA*-specific role, while sRNA-2 may affect genes that are involved in normal physiology. However, of note is our observation that sRNA-2 is located within the “extended pathogenicity island”, defined as containing all of the genes on pXO1 that are known to be related to virulence. This suggests a potential common ancestry for acquisition of the pathogenicity island containing sRNA-2 within it. Characterizing the specific functions of these two sRNAs will require further experimentation.

An interesting finding was that many of the genes that are regulated by AtxA appear to be clustered in coordinated expression groups on pXO1, a finding which may give insight into the regulatory function of AtxA. For example, *pXO1_0123* appeared to be co-transcribed (near equal read numbers) with the adjacent *bslA* (*pXO1_0124*). However, their staircase-like pattern of gene expression [58] indicates that each of these genes may also have its own promoter. This could suggest that AtxA binds independently to each putative promoter to induce or modulate gene expression. Although this is possible, the clustering of AtxA-regulated genes suggests that AtxA may instead act in a more a global fashion, perhaps by simultaneously increasing the probability of RNA polymerase binding to both promoters. One plausible mechanism previously proposed is that AtxA could act as, or interact with, a nucleoid-associated protein to alter the local DNA topology of certain genomic regions [59]. Previous studies have found that the three AtxA-regulated toxin-encoding genes have distinct DNA curvature patterns, which is consistent with this hypothesis [48].

## Conclusions

Due to the factorial design of our experiment, the resulting data permit inferences to be made regarding the contributions of both genotype and environment to the responses of genes regulated by AtxA, CO_2_, or both. The results are consistent with a model in which AtxA is essential to stimulate expression greater than baseline and in which environments enriched in CO_2_ act to modulate the degree of that stimulation. Consistent with other studies, we found that the expression of *atxA* itself is not increased as significantly as the genes it stimulates, if at all, when *B. anthracis* is grown in environments containing increased concentrations of CO_2_[11,12,16]. AtxA is regulated downstream of transcription by a number of CO_2_-related processes [20,22-24,60], and our data provide further evidence that this regulation alone is sufficient to account for its transcriptional effects on virulence-related genes. Our data also provide insights into two novel small RNAs that are strongly associated with the combined presence of CO_2_ and AtxA. Further experiments into the action of these small RNAs may offer additional insights into the mechanism of the interaction between CO_2_ and AtxA. Finally, we define a number of unannotated genes whose expression is coordinated with known virulence-related genes. The components of the AtxA regulatory pathway that we describe here and their interactions with CO_2_ represent molecular targets for specific inhibitory interventions aimed at decreasing the pathogenesis of *B. anthracis*.

### Availability of supporting data

The Illumina reads have been deposited to the Small Read Archive repository at NCBI (http://www.ncbi.nlm.nih.gov/sra) and they are available under study accession number SRP015914.

## Abbreviations

AtxA: Anthrax Toxin Activator; PA: Protective antigen; EF: Edema factor; LF: Lethal factor; sRNA: Small RNA; WT: Wildtype.

## Competing interests

The authors declare that they have no competing interests.

## Authors’ contributions

AM, AP, SP, and SL conceived and designed the experiments. AM and AP generated the AtxA deletion strain. AM, AP, and IS carried out the isolation and purification of the RNA. SR prepared samples for RNA and small RNA-Sequencing. SA performed ribosomal RNA depletion. CM performed read mapping, differential analysis, and small RNA analysis. AM, AP, IS, CM, SP, and SL analyzed the RNA sequencing data. AM, SP, and KV designed and carried out the qPCR validation experiments. AM, AP, IS, CM, SR, SP, and SL drafted and edited the manuscript. All authors read and approved the manuscript.

## Supplementary Material

Additional file 1: Table S1 Plasmids, strains, and primers used in this study. **Table S2.** Primers and probes used for qPCR. **Table S3.** Genes having increased expression in conditions of ΔAtxA and air (total =17). **Table S4.** Genes having increased expression in conditions of ΔAtxA and CO2 (total = 1). **Table S5.** Genes having increased expression in conditions of wildtype and air (total = 23). **Table S6.** Genes having increased expression in wildtype genotypes (total = 95). **Table S7.** Genes having increased expression in ΔAtxA genotypes (total = 44). **Table S8.** Genes having increased expression in environments of CO2 (total = 99). **Table S9.** Genes having increased expression in environments of air (total = 219). **Figure S1.** Representative growth curves of wildtype and ΔAtxA strains in air and CO2. **Figure S2.** Comparison of RNA-seq to qPCR expression data of seven genes from each condition. Click here for file
