# Supplementary material for: Transcriptome analysis identifies Bacillus anthracis genes that respond to CO2 through an AtxA-dependent mechanism
Source: BMC Genomics. 2014 Mar 25;15:229. doi: 10.1186/1471-2164-15-229 (PMC3987803; doi:10.1186/1471-2164-15-229)
Supplement: Additional file 1: Table S1 — Plasmids, strains, and primers used in this study. Table S2. Primers and probes used for qPCR. Table S3. Genes having increased expression in conditions of ΔAtxA and air (total =17). Table S4. Genes having increased expression in conditions of ΔAtxA and CO2 (total = 1). Table S5. Genes having increased expression in conditions of wildtype and air (total = 23). Table S6. Genes having increased expression in wildtype genotypes (total = 95). Table S7. Genes having increased expression in ΔAtxA genotypes (total = 44). Table S8. Genes having increased expression in environments of CO2 (total = 99). Table S9. Genes having increased expression in environments of air (total = 219). Figure S1. Representative growth curves of wildtype and ΔAtxA strains in air and CO2. Figure S2. Comparison of RNA-seq to qPCR expression data of seven genes from each condition. [file 1471-2164-15-229-S1.pdf]

Supplementary Tables and Figures

Supplementary Table 1
Plasmids, strains, and primers used in this study.

**Plasmids**	**Relevant Characteristics**	**Reference**
pSC	Temperature-sensitive vector to insert homologous DNA	[1]
pCrePAS	Temperature-sensitive vector expressing Cre recombinase	[1]
pSC-AtxA-L	From pSC, with DNA fragment upstream of the AtxA ORF inserted into XhoI and SpeI sites	This work
pSC-AtxA-R	From pSC, with DNA fragment downstream of the AtxA ORF inserted into XhoI and SpeI sites	This work
pIU68	AtxA complement vector	[2]
**Strains**	**Relevant Characteristics**	
Ames 35	*B. anthracis* Ames pXO1+ pXO2- ANR-1 strain	[3], [4]*
Ames 35 ΔAtxA	Ames 35 with *loxP* site replacing *atxA* ORF	This work
Ames 35 ΔAtxA pIU68	Ames 35 ΔAtxA transformed with pIU68	This work
**Oligonucleotides**	**Sequence**	
AtxA LF	CCTCGAGACCAAAAATTTCAAGGTGAT	
AtxA LR	AACTAGTCGGTGTTAGCATGTCTATAATTGA	
AtxA RF	CCTCGAGAAAAGATAATATAAATGCCC	
AtxA RR	AACTAGTAGTGGGTACGACGATTCCAA	
AtxA Int RF	AAAGAGGAAAAGGAGAGATT	
AtxA Int RR	CCCATGAGAATACTTTTCAA	
AtxA Fin F	ACATAAGTATCGTTTTGTTGCTTCC	
AtxA Fin R	CCAAGTAAATAAGAAACCGCCAAAT	

* The Ames 35 strain designation is our lab's name for Ames cured of pXO2 and retaining pXO1. This strain is equivalent (and may be identical) to the strain ANR-1 that is mentioned in the text (but not the strain table) of Green et al. [3]. The equivalence of Ames 35 to ANR-1 was stated in the strain table of Pomerantsev et al. [4].

Reference List

[1] Pomerantsev AP, Camp A, Leppla SH: A new minimal replicon of *Bacillus anthracis* plasmid pXO1. J Bacteriol 6-5-2009;191:5134-5146.
[2] Uchida I, Hornung JM, Thorne CB, Klimpel KR, Leppla SH: Cloning and characterization of a gene whose product is a trans-activator of anthrax toxin synthesis. J Bacteriol 1993;175:5329-5338.
[3] Green BD, Battisti L, Koehler TM, Thorne CB, Ivins BE: Demonstration of a capsule plasmid in *Bacillus anthracis*. Infect Immun 1985;49:291-297.
[4] Pomerantsev AP, Sitaraman R, Galloway CR, Kivovich V, Leppla SH: Genome engineering in *Bacillus anthracis* using Cre recombinase. Infect Immun 2006;74:682-693.

Supplementary Table 2.
Primers and probes used for qPCR

Gene (NCBI ID)		Primer/Probe Sequences
Aspartokinase (GBAA3936)	Forward primer	5' GTGCCGCTTCGTGTACGTT 3'
	Reverse primer	5' AATACCTGTAACCGGACGTTCTTC 3'
	Probe	5' CAL Fluor Gold 540 ATCCTGCCCTTTCGTAGCACCATCGTA BHQ1 3'
Germination protein gerN (GBAA1639)	Forward primer	5' CCATTCGTAGGTGGTTACGTTTC 3'
	Reverse primer	5' ACACTTGTCGCACATAGAAGTAATCCT 3'
	Probe	5' FAM ACAGCATTTCCTTGTTCCATTCCCATAACA BHQ1 3'
S-layer protein (GBAA_pXO1_0124)	Forward primer	5' AATAGTTCCAGATCAAATGGTCCC 3'
	Reverse primer	5' TCCCTTGTGACTGAATCACCTAATC 3'
	Probe	5' FAM TTCTGGACGTTCCATCAAATCATTGGG BHQ1 3'
S-layer protein EA1 (GBAA0887)	Forward primer	5' GTACTGGTGGTAAATGGGAGCC 3'
	Reverse primer	5' GAAACTTTCACATCAGTTACATACGCT 3'
	Probe	5' FAM CGTGCAGAAGCAGCTCAATTTATCGCAT BHQ1 3'
Universal stress protein family (GBAA4875)	Forward primer	5' AATTATGTGTGGTGCAACTGGTTT 3'
	Reverse primer	5' GAACAACAAGGACATCGCATTTC 3'
	Probe	5' FAM CGCTACCAATTAGGAAACGCTCAACAGCAT BHQ1 3'
Unknown sRNA (sRNA-1)	Forward primer	5' AGACTACATACAGCTATCGATTGGAAATT 3'
	Reverse primer	5' TTTACCTCTGTTAAGTCCAGTATCTATTCTATTC 3'
	Probe	5' FAM CATTCCCATTTTATTGTGTCCCATCAGCCT BQH1 3'
Unknown putative transporter (GBAA2840)	Forward primer	5' AATTCTCAAAAAACGACTCCAGATTC 3'
	Reverse primer	5' ACCACCAAGACCACCTAGTAATAAACC 3'
	Probe	5' FAM AAAACGAAAGCAACAAACACAGCACCAAAA BHQ1 3'
Unknown sRNA (sRNA-2)	Forward primer	5' AGTTCAAGAGTTACTGAAACTAGCCATT3'
	Reverse primer	5' AGAACCAAATTGAATCACGTTATAATTC 3'
	Probe	5' FAM TCCTAATATGGAGAAAAGATGGCTATCGCTTCTTCACCT BHQ1 -3'

Supplementary Table 3.
Genes having increased expression in conditions of ΔAtxA and air (total =17)

Locus Tag	Protein Product (NCBI)	Protein Annotation	P-Value	Fold-Change
GBAA0631	YP_017259.1	PTS system, trehalose-specific IIBC component (EC 2.7.1.69)	<0.0001	241.4
GBAA0730	YP_017363.1	Glycine oxidase (EC 1.4.3.19)	0.0076	4.1
GBAA0733	YP_017366.1	Molybdopterin biosynthesis MoeB protein	0.0007	4.0
GBAA1158	YP_017774.1	Ferrochelatase (EC 4.99.1.1)	0.0006	72.9
GBAA2125	YP_018768.1	Respiratory nitrate reductase alpha chain (EC 1.7.99.4)	<0.0001	8.4
GBAA2126	YP_018769.1	Respiratory nitrate reductase beta chain (EC 1.7.99.4)	<0.0001	9.2
GBAA2127	YP_018770.1	Respiratory nitrate reductase delta chain (EC 1.7.99.4)	<0.0001	8.2
GBAA2128	YP_018771.1	Respiratory nitrate reductase gamma chain (EC 1.7.99.4)	0.0093	7.8
GBAA2134	YP_018777.1	Molybdopterin biosynthesis MoeB protein	<0.0001	8.6
GBAA2135	YP_018778.1	Molybdopterin biosynthesis MoeA protein	<0.0001	7.0
GBAA2136	YP_018779.1	Molybdopterin converting factor, large subunit	<0.0001	9.3
GBAA2137	YP_018780.1	Molybdopterin converting factor, small subunit	0.0011	12.0
GBAA2138	YP_018781.1	Nitrite extrusion protein	<0.0001	7.0
GBAA2367	YP_019011.1	Oxalate/formate antiporter	<0.0001	11.4
GBAA3317	YP_019951.2	Amino acid permease	0.0025	12.2
GBAA3497	YP_020132.1	Hydroxylamine reductase (EC 1.7.99.1)	<0.0001	6.2
GBAA5273	YP_021930.1	Hypothetical protein	<0.0001	4.1

This table reports genes with a significant interaction ($p<0.01$) between genotype and environment as well as increased expression in the ΔAtxA/air condition compared to the WT/CO_2 condition (fold-change > 4).

Supplementary Table 4.
Genes having increased expression in conditions of ΔAtxA and CO_2 (total = 1)

Locus Tag	Protein Product (NCBI)	Protein Annotation	P-Value	Fold-Change
GBAA1946	YP_018590.1	Transport ATP-binding protein cydC	0.0044	20.2

This table reports genes with a significant interaction ($p<0.01$) between genotype and environment as well as increased expression in the ΔAtxA/CO_2 condition compared to the WT/air condition (fold-change > 4).

Supplementary Table 5.
Genes having increased expression in conditions of wildtype and air (total = 23)

Locus Tag	Protein Product (NCBI)	Protein Annotation	P-Value	Fold-Change
GBAA_pXO1_0124	YP_016455.2	S-layer protein	<0.0001	16.3
GBAA_pXO1_0125	YP_016456.2	Phosphatidylglycerophosphatase B (EC 3.1.3.27)	<0.0001	4.0
GBAA_pXO1_0151	YP_016482.2	Hypothetical protein	<0.0001	Inf
GBAA_pXO1_0153	YP_016484.2	Hypothetical protein	<0.0001	Inf
sRNA-1		sRNA-1	<0.0001	33.6
sRNA-2		sRNA-2	<0.0001	26.8
GBAA1158	YP_017774.1	Ferrochelatase (EC 4.99.1.1)	0.0006	14.4
GBAA2125	YP_018768.1	Respiratory nitrate reductase alpha chain (EC 1.7.99.4)	<0.0001	11.6
GBAA2126	YP_018769.1	Respiratory nitrate reductase beta chain (EC 1.7.99.4)	<0.0001	11.8
GBAA2127	YP_018770.1	Respiratory nitrate reductase delta chain (EC 1.7.99.4)	<0.0001	12.9
GBAA2128	YP_018771.1	Respiratory nitrate reductase gamma chain (EC 1.7.99.4)	0.0093	15.5
GBAA2134	YP_018777.1	Molybdopterin biosynthesis MoeB protein	<0.0001	13.6
GBAA2135	YP_018778.1	Molybdopterin biosynthesis MoeA protein	<0.0001	10.2
GBAA2136	YP_018779.1	Molybdopterin converting factor, large subunit	<0.0001	11.0
GBAA2137	YP_018780.1	Molybdopterin converting factor, small subunit	0.0011	13.5
GBAA2138	YP_018781.1	Nitrite extrusion protein	<0.0001	11.5
GBAA2143	YP_018786.1	Sirohydrochlorin cobaltochelatase (EC 4.99.1.3)	<0.0001	4.4
GBAA2144	YP_018787.1	Uroporphyrin-III C-methyltransferase (EC 2.1.1.107) / Uroporphyrinogen-III synthase (EC 4.2.1.75)	<0.0001	4.1
GBAA2145	YP_018788.1	Nitrite reductase [NAD(P)H] small subunit (EC 1.7.1.4)	0.0017	4.5
GBAA2146	YP_018789.1	Nitrite reductase [NAD(P)H] large subunit (EC 1.7.1.4)	0.0023	4.3
GBAA2367	YP_019011.1	Oxalate/formate antiporter	<0.0001	19.9
GBAA3497	YP_020132.1	Hydroxylamine reductase (EC 1.7.99.1)	<0.0001	16.5
GBAA3560	YP_020193.1	Glycerophosphoryl diester phosphodiesterase (EC 3.1.4.46)	0.0029	7.3

This table reports genes with a significant interaction ($p<0.01$) between genotype and environment as well as increased expression in the WT/air condition compared to the ΔAtxA/CO_2 condition (fold-change > 4).

Supplementary Table 6.
Genes having increased expression in wildtype genotypes (total = 95)

Locus Tag	Protein Product (NCBI)	Protein Annotation	P-Value	Fold-Change
GBAA_pXO1_0123	YP_016454.2	Hypothetical protein	<0.0001	42.0
GBAA_pXO1_0124	YP_016455.2	S-layer protein	<0.0001	123.1
GBAA_pXO1_0125	YP_016456.2	Phosphatidylglycerophosphatase B (EC 3.1.3.27)	<0.0001	22.7
GBAA_pXO1_0137	YP_016468.2	Hypothetical protein	<0.0001	22.7
GBAA_pXO1_0145	YP_016476.2	Putative integral membrane protein	<0.0001	4.3
GBAA_pXO1_0146	YP_016477.2	Anthrax toxins expression trans-acting positive regulator	<0.0001	110.5
GBAA_pXO1_0151	YP_016482.2	Hypothetical protein	<0.0001	143.9
GBAA_pXO1_0152	YP_016483.2	Hypothetical protein	<0.0001	192.8
GBAA_pXO1_0153	YP_016484.2	Hypothetical protein	<0.0001	71.9
GBAA_pXO1_0164	YP_016495.2	Protective surface antigen	<0.0001	72.2
GBAA_pXO1_0165	YP_016496.2	Hypothetical protein	<0.0001	45.1
GBAA_pXO1_0166	YP_016497.2	Transcriptional repressor pagR	<0.0001	58.0
GBAA_pXO1_0171	YP_016502.2	Ribonuclease domain protein	0.0004	8.9
GBAA_pXO1_0172	YP_016503.2	Lethal factor precursor (EC 3.4.24.-)	<0.0001	9.5
GBAA_pXO1_0205	YP_022466.1	Response regulator aspartate phosphatase	0.0024	3.1
sRNA-1		sRNA-1	0.0009	315.4
sRNA-2		sRNA-2	0.0004	308.0
GBAA0170	YP_016777.1	Hypothetical protein	0.0002	3.1
GBAA0171	YP_016779.1	Hypothetical Protein	0.0001	3.0
GBAA0238	YP_016844.1	Hypothetical Protein	0.0079	2.6
GBAA0361	YP_016980.1	Polyketide cyclase family protein	0.0053	2.5
GBAA0553	YP_017175.1	Acetyltransferase (EC 2.3.1.-)	<0.0001	3.9
GBAA0793	YP_017427.1	PTS system, diacetylchitobiose-specific IIC component	0.0003	6.3
GBAA0795	YP_052611.1	outer surface protein	0.0027	5.5
GBAA0864	YP_017501.1	Hypothetical protein	0.0085	3.0
GBAA0887	YP_017522.1	S-layer protein EA1 precursor	<0.0001	5.8
GBAA0918	YP_017555.1	Hypothetical protein	0.0001	4.5
GBAA0936	YP_017571.1	Hypothetical membrane associated protein	0.0003	7.0
GBAA0956	YP_017588.1	Hypothetical protein	0.0056	3.1
GBAA1061	YP_017686.1	Hypothetical protein	0.0098	2.4
GBAA1127	YP_017747.1	S-layer homology domain	<0.0001	3.9
GBAA1363	YP_017982.1	Hypothetical protein	<0.0001	7.0
GBAA1378	YP_018000.1	CAAX amino terminal protease family	0.0096	2.3
GBAA1480	YP_018102.1	Hypothetical protein	<0.0001	3.2
GBAA1579	YP_018202.1	Hypothetical protein	<0.0001	5.4

GBAA1816	YP_018453.1	Phosphohydrolase (MutT/nudix family protein)	0.0079	2.5
GBAA2000	YP_018647.1	Transition state regulator	<0.0001	3.6
GBAA2301	YP_018949.1	Hypothetical cytosolic protein	0.0063	2.6
GBAA2411	YP_019053.1	Acetyltransferase (EC 2.3.1.-)	0.0003	3.6
GBAA2467	YP_019106.2	Hypothetical membrane spanning protein	0.0015	2.7
GBAA2485	YP_019124.1	Methyltransferase (EC 2.1.1.-)	0.0009	2.5
GBAA2527	YP_019165.1	Hypothetical protein	0.0009	4.0
GBAA2534	YP_019173.1	Ribosomal-protein-serine acetyltransferase (EC 2.3.1.-)	0.0003	2.8
GBAA2547	YP_019185.1	Acyl-CoA dehydrogenase, short-chain specific (EC 1.3.99.2)	<0.0001	4.0
GBAA2548	YP_019186.1	Propionyl-CoA carboxylase biotin-containing subunit (EC 6.4.1.3)	0.0007	3.1
GBAA2550	YP_019188.1	Hydroxymethylglutaryl-CoA lyase (EC 4.1.3.4)	0.0028	3.0
GBAA2552	YP_019190.1	Propionyl-CoA carboxylase beta chain (EC 6.4.1.3)	0.0085	2.7
GBAA2673	YP_019314.1	Chitosanase (EC 3.2.1.132)	0.0085	2.6
GBAA2781	YP_019423.1	Phosphohydrolase (MutT/nudix family protein)	0.0002	4.1
GBAA2783	YP_019425.2	Hypothetical protein	0.0001	3.6
GBAA2816	YP_052628.1	N-METHYL-TRANSFERASE	0.0035	2.7
GBAA2956	YP_019600.1	Chorismate synthase (EC 4.2.3.5)	0.0074	2.7
GBAA3011	YP_019655.1	Glyoxalase family protein	0.004	2.9
GBAA3043	YP_019684.1	Hypothetical protein	0.0041	4.7
GBAA3046	YP_019687.1	Hypothetical Membrane Spanning Protein	0.0027	2.8
GBAA3047	YP_019688.1	ABC transporter permease protein	0.0003	3.3
GBAA3200	YP_019841.2	Hypothetical protein	0.0068	2.5
GBAA3227	YP_019866.1	Hypothetical Protein	<0.0001	7.6
GBAA3283	YP_019919.1	Transcriptional regulator, LacI family	0.0033	3.1
GBAA3411	YP_020046.1	Integral membrane protein	0.0055	2.5
GBAA3498	YP_020133.1	Hypothetical protein	0.0049	2.6
GBAA3504	YP_020138.1	LSU ribosomal protein L12P (L7/L12)	0.0003	2.9
GBAA3519	YP_020154.1	Hypothetical protein	<0.0001	5.4
GBAA3529	YP_020163.1	Hypothetical protein	<0.0001	3.2
GBAA3560	YP_020193.1	Glycerophosphoryl diester phosphodiesterase (EC 3.1.4.46)	<0.0001	5.2
GBAA3683	YP_020316.1	Hypothetical protein	0.0002	3.2
GBAA3691	YP_020324.1	Hypothetical protein	0.0002	3.1
GBAA3692	YP_020325.1	Hypothetical Protein	<0.0001	3.3
GBAA3694	YP_020327.1	Hypothetical protein	0.0002	9.9
GBAA3804	YP_020437.1	Hypothetical protein	0.0015	3.2
GBAA3860	YP_020498.1	Copper chaperone copZ	0.0034	3.0
GBAA3874	YP_020514.1	Hypothetical protein	0.0002	2.9

GBAA4065	YP_020708.1	Hypothetical protein	0.0007	3.2
GBAA4211	YP_020853.1	Lipoprotein	0.0002	2.9
GBAA4242	YP_020884.1	Hypothetical protein	<0.0001	3.6
GBAA4345	YP_020990.1	Tyrosine transporter	0.0033	2.6
GBAA4420	YP_022691.1	Hypothetical protein	0.0056	3.2
GBAA4581	YP_021226.1	Hypothetical protein	0.0007	3.0
GBAA4582	YP_021227.1	Hypothetical protein	<0.0001	3.6
GBAA4739	YP_021387.1	Stress-responsive transcriptional regulator PspC	0.0082	3.3
GBAA4927	YP_021570.1	Hypothetical protein	<0.0001	3.0
GBAA4928	YP_021571.1	Hypothetical protein	<0.0001	3.4
GBAA4934	YP_021579.1	Hypothetical protein	0.0082	2.5
GBAA4941	YP_021587.1	Hypothetical protein	0.0063	3.4
GBAA5015	YP_021667.1	Hypothetical membrane spanning protein	0.0011	3.0
GBAA5018	YP_022711.1	Hypothetical protein	0.0016	2.7
GBAA5020	YP_022712.1	Hypothetical protein	0.0006	2.9
GBAA5045	YP_021693.1	Hypothetical protein	<0.0001	3.9
GBAA5072	YP_021721.1	Hypothetical protein	0.0012	3.1
GBAA5183	YP_021838.1	CAAX amino terminal protease family	0.0025	3.3
GBAA5187	YP_021842.1	Hypothetical protein	0.0056	2.4
GBAA5202	YP_021858.1	Hypothetical protein	0.0001	3.6
GBAA5203	YP_021859.1	Phosphoglycerate mutase family protein	0.0076	2.5
GBAA5258	YP_021914.2	Collagen adhesion protein	0.0034	2.6
GBAA5351	YP_022009.1	Phage protein	0.0076	2.5

This table reports genes with a significant difference in expression ($p<0.01$) between genotypes, as well as increased expression in the WT strain as compared to the ΔAtxA mutants strain (fold-change > 2).

Supplementary Table 7.
Genes having increased expression in ΔAtxA genotypes (total = 44).

Locus Tag	Protein Product (NCBI)	Protein Annotation	P-Value	Fold-Change
GBAA0040	YP_016644.1	Sporulation-specific protease YabG	0.0024	2.4
GBAA0344	YP_016960.1	Peroxiredoxin reductase (NAD(P)H) (EC 1.8.1.-) / NADH oxidase H2O2-forming (EC 1.6.3.-)	0.0009	2.5
GBAA0349	YP_016965.1	Ferrichrome transport system permease protein fhuG	0.0028	2.3
GBAA0350	YP_016966.1	Ferrichrome transport system permease protein fhuB	0.0063	2.3
GBAA0383	YP_017003.1	Hydroxymethylpyrimidine transport ATP-binding protein	0.0066	2.6
GBAA0495	YP_017114.1	Integral membrane protein	0.0002	3.5
GBAA0534	YP_017153.1	ABC transporter permease protein	0.0008	2.6
GBAA0615	YP_017241.1	Fe3+-siderophore binding protein	<0.0001	3.4
GBAA0617	YP_017244.1	Fe3+-siderophore transport system permease protein	0.0012	2.5
GBAA0631	YP_017259.1	PTS system, trehalose-specific IIBC component (EC 2.7.1.69)	<0.0001	103.8
GBAA0745	YP_017377.1	Phospholipase (EC 3.1.-.-)	0.004	2.7
GBAA0802	YP_017440.1	Branched-chain amino acid transport system carrier protein	0.0004	3.9
GBAA0829	YP_017467.1	Transporter, Drug/Metabolite Exporter family	0.0018	5.5
GBAA0845	YP_017484.1	Amino acid permease	0.0085	2.6
GBAA0981	YP_017610.1	S-layer protein / Peptidoglycan endo-beta-N-acetylglucosaminidase (EC 3.2.1.-)	0.0015	2.3
GBAA1088	YP_017711.1	Metal-dependent hydrolase (EC 3.-.-.-)	0.0045	2.3
GBAA1452	YP_018076.2	Na+/H+ antiporter nhaC	0.0001	2.8
GBAA1943	YP_018587.1	Cytochrome d ubiquinol oxidase subunit I (EC 1.10.3.-)	<0.0001	3.3
GBAA1944	YP_018588.1	Cytochrome d ubiquinol oxidase subunit II (EC 1.10.3.-)	<0.0001	3.0
GBAA1945	YP_018589.1	Transport ATP-binding protein cydD	<0.0001	3.0
GBAA1946	YP_018590.1	Transport ATP-binding protein cydC	<0.0001	2.5
GBAA2016	YP_018663.1	Luciferase-like monooxygenase (EC 1.14.-.-)	<0.0001	3.7
GBAA2370	YP_019014.1	2,3-dihydroxybenzoate-AMP ligase (EC 2.7.7.58)	0.0016	3.3
GBAA3317	YP_019951.2	Amino acid permease	0.0034	4.0
GBAA3429	YP_020064.1	Gluconate permease	0.0068	2.3
GBAA3431	YP_020066.1	6-phosphogluconate dehydrogenase (EC 1.1.1.44)	0.0079	2.3
GBAA3438	YP_020073.1	Quinone oxidoreductase (EC 1.6.5.5)	0.0031	2.2
GBAA3439	YP_022642.1	Hypothetical protein	0.0003	2.7
GBAA3538	YP_020172.1	Hypothetical protein	0.0006	2.8
GBAA3595	YP_020230.2	BNR-repeat containing protein	0.0082	2.9
GBAA3631	YP_052641.1	Formate dehydrogenase alpha chain (EC 1.2.1.2)	<0.0001	2.9
GBAA4145	YP_020792.1	Hypothetical cytosolic protein	0.0034	2.6
GBAA4146	YP_020793.1	Hypothetical cytosolic protein	0.003	2.5
GBAA4595	YP_021241.1	Ferrichrome transport ATP-binding protein fhuC	0.0012	4.5

GBAA4652	YP_021299.1	Ferrichrome-binding protein	<0.0001	7.9
GBAA4694	YP_021342.1	Delta-aminolevulinic acid dehydratase (EC 4.2.1.24)	0.0027	2.3
GBAA5063	YP_022716.1	Hypothetical protein	0.0048	3.6
GBAA5064	YP_021713.1	Ferrous iron transport protein B	0.0002	4.2
GBAA5281	YP_021938.1	Hypothetical cytosolic protein	0.0066	3.9
GBAA5301	YP_021959.1	Amino acid carrier protein alsT homolog	<0.0001	5.1
GBAA5328	YP_021986.1	Ferric anguibactin transport system permease protein fatC	<0.0001	2.8
GBAA5329	YP_021987.1	Ferric anguibactin transport system permease protein fatD	0.0002	2.6
GBAA5330	YP_021988.2	Ferric anguibactin-binding protein	<0.0001	3.8
GBAA5616	YP_022288.1	Hypothetical protein	0.0085	2.6

This table reports genes with a significant difference in expression ($p<0.01$) between genotypes, as well as increased expression in the ΔAtxA mutants strain as compared to the WT strain (fold-change > 2).

Supplementary Table 8.
Genes having increased expression in environments of CO_2 (total = 99)

Locus Tag	Protein Product (NCBI)	Protein Annotation	P-Value	Fold-Change
GBAA_pXO1_0123	YP_016454.2	Hypothetical protein	<0.0001	21.3
GBAA_pXO1_0124	YP_016455.2	S-layer protein	0.0040	16.7
GBAA_pXO1_0125	YP_016456.2	Phosphatidylglycerophosphatase B (EC 3.1.3.27)	0.0005	8.3
GBAA_pXO1_0137	YP_016468.2	Hypothetical protein	<0.0001	9.9
GBAA_pXO1_0148	YP_016479.2	Histidine protein kinase	0.0071	2.2
GBAA_pXO1_0151	YP_016482.2	Hypothetical protein	<0.0001	46.6
GBAA_pXO1_0152	YP_016483.2	Hypothetical protein	<0.0001	45.4
GBAA_pXO1_0153	YP_016484.2	Hypothetical protein	<0.0001	18.1
GBAA_pXO1_0164	YP_016495.2	Protective surface antigen	<0.0001	47.6
GBAA_pXO1_0165	YP_016496.2	Hypothetical protein	<0.0001	27.5
GBAA_pXO1_0166	YP_016497.2	Transcriptional repressor pagR	<0.0001	36.9
GBAA_pXO1_0171	YP_016502.2	Ribonuclease domain protein	<0.0001	9.8
GBAA_pXO1_0172	YP_016503.2	Lethal factor precursor (EC 3.4.24.-)	<0.0001	8.4
GBAA0141	YP_016746.1	Cobalt transport protein cbiQ	0.0002	2.2
GBAA0164	YP_016771.1	6-phosphogluconate dehydrogenase (EC 1.1.1.44)	<0.0001	2.2
GBAA0239	YP_016845.1	Hypothetical Protein	0.0011	2.1
GBAA0366	YP_016985.1	Fatty acid desaturase (EC 1.14.99.-)	<0.0001	2.4
GBAA0406	YP_017026.1	Hypothetical cytosolic protein	0.0004	2.4
GBAA0430	YP_017055.1	Hypothetical protein	0.0001	2.3
GBAA0495	YP_017114.1	Integral membrane protein	0.0007	2.7
GBAA0556	YP_017178.1	Hypothetical protein	<0.0001	3.8
GBAA0557	YP_017179.1	Flotillin	<0.0001	3.6
GBAA0793	YP_017427.1	PTS system, diacetylchitobiose-specific IIC component	<0.0001	5.5
GBAA0795	YP_052611.1	outer surface protein	<0.0001	5.7
GBAA0811	YP_017449.1	Hypothetical protein	0.0062	2.2
GBAA0832	YP_017470.1	Quaternary ammonium compound-resistance protein	<0.0001	10.3
GBAA0833	YP_017471.1	Quaternary ammonium compound-resistance protein	<0.0001	7.8
GBAA0958	YP_017590.1	Transcriptional regulator, MarR family	<0.0001	3.1
GBAA0959	YP_017591.1	CDP-diacylglycerol--serine O-phosphatidyltransferase (EC 2.7.8.8)	<0.0001	2.9
GBAA0960	YP_017592.1	ATP synthase protein I	<0.0001	2.9
GBAA1178	YP_017791.1	Hypothetical protein	0.008	2.3
GBAA1192	YP_017805.1	Oligopeptide transport system permease protein oppB	<0.0001	2.4
GBAA1193	YP_017806.1	Oligopeptide transport system permease	<0.0001	2.8

		protein oppC		
GBAA1194	YP_017807.1	Oligopeptide transport ATP-binding protein oppD	<0.0001	2.1
GBAA1195	YP_017808.1	Oligopeptide transport ATP-binding protein oppF	<0.0001	2.7
GBAA1363	YP_017982.1	Hypothetical protein	0.0001	2.8
GBAA1480	YP_018102.1	Hypothetical protein	<0.0001	2.0
GBAA1639	YP_018274.1	Na+/H+ antiporter NapA (inosine-dependent germination)	<0.0001	16.8
GBAA1815	YP_018452.1	Hypothetical protein	<0.0001	2.7
GBAA1822	YP_018459.1	Carbon starvation protein A	<0.0001	2.8
GBAA1826	YP_018464.1	3-oxoacyl-[acyl-carrier-protein] synthase III (EC 2.3.1.41)	<0.0001	2.9
GBAA1892	YP_018536.1	Deoxyribose-phosphate aldolase (EC 4.1.2.4)	<0.0001	2.6
GBAA1893	YP_018537.1	Nucleoside permease nupC	<0.0001	3.2
GBAA1894	YP_018538.1	Pyrimidine-nucleoside phosphorylase (EC 2.4.2.2)	<0.0001	2.5
GBAA1895	YP_018539.1	Cytidine deaminase (EC 3.5.4.5)	<0.0001	2.6
GBAA1943	YP_018587.1	Cytochrome d ubiquinol oxidase subunit I (EC 1.10.3.-)	<0.0001	4.5
GBAA1944	YP_018588.1	Cytochrome d ubiquinol oxidase subunit II (EC 1.10.3.-)	<0.0001	3.9
GBAA1945	YP_018589.1	Transport ATP-binding protein cydD	<0.0001	4.2
GBAA1946	YP_018590.1	Transport ATP-binding protein cydC	<0.0001	3.3
GBAA1984	YP_018628.1	Acyl carrier protein	<0.0001	2.4
GBAA1985	YP_018629.1	Hypothetical Protein	<0.0001	3.0
GBAA1986	YP_018630.1	Hypothetical cytosolic protein	<0.0001	4.4
GBAA2061	YP_018702.1	CBS domain containing protein	<0.0001	2.2
GBAA2539	YP_019178.1	phosphoesterase (EC 3.1.-.-)	<0.0001	2.7
GBAA2839	YP_019482.1	Hypothetical protein	<0.0001	6.9
GBAA2840	YP_019483.1	Transporter	<0.0001	8.6
GBAA2841	YP_019484.1	Hypothetical Protein	<0.0001	2.9
GBAA2918	YP_019560.1	Hypothetical protein	0.008	2.3
GBAA2996	YP_019639.1	PEPTIDASE FAMILY M20	<0.0001	4.7
GBAA3258	YP_019895.1	Macrolide-efflux protein	<0.0001	3.6
GBAA3445	YP_020078.1	Hypothetical protein	0.0002	2.2
GBAA3645	YP_020278.1	Oligopeptide-binding protein oppA	<0.0001	3.0
GBAA3988	YP_020628.1	Acyl carrier protein	0.0002	2.1
GBAA3989	YP_020629.1	3-oxoacyl-[acyl-carrier protein] reductase (EC 1.1.1.100)	<0.0001	2.2
GBAA4060	YP_020704.1	Acetyltransferase (EC 2.3.1.-)	<0.0001	2.8
GBAA4242	YP_020884.1	Hypothetical protein	<0.0001	2.9
GBAA4265	YP_020907.1	Hypothetical protein	0.001	2.3
GBAA4266	YP_020908.1	Hypothetical protein	0.0084	2.3
GBAA4337	YP_020983.1	Biotin synthesis protein bioC	0.0008	2.4

GBAA4339	YP_020985.1	8-amino-7-oxononanoate synthase (EC 2.3.1.47)	<0.0001	3.4
GBAA4340	YP_020986.1	Dethiobiotin synthetase (EC 6.3.3.3)	0.0002	3.1
GBAA4341	YP_020987.1	Adenosylmethionine-8-amino-7-oxononanoate aminotransferase (EC 2.6.1.62)	<0.0001	2.9
GBAA4434	YP_021077.1	Transcriptional regulator, TetR family	<0.0001	3.0
GBAA4435	YP_021078.1	Quaternary ammonium compound-resistance protein	<0.0001	3.4
GBAA4437	YP_021080.1	Hypothetical protein	0.0039	2.5
GBAA4473	YP_021116.1	Hypothetical protein	0.0007	2.3
GBAA4490	YP_021134.1	LSU ribosomal protein L33P	<0.0001	2.3
GBAA4494	YP_021138.1	Phosphate transport system permease protein pstA	0.0062	2.1
GBAA4495	YP_021139.1	Phosphate transport system permease protein pstC	0.0052	2.1
GBAA4514	YP_021159.1	Membrane-attached cytochrome c550	0.0069	2.3
GBAA4617	YP_021265.1	Hypothetical Protein	<0.0001	2.3
GBAA4751	YP_021400.1	Thioesterase (EC 3.1.2.-)	<0.0001	2.3
GBAA4865	YP_022705.1	Hypothetical protein	<0.0001	5.3
GBAA4875	YP_021517.1	Universal stress protein family	<0.0001	2.8
GBAA4967	YP_021614.2	CarD-like transcriptional regulator	0.0004	2.2
GBAA5051	YP_021699.1	Cytochrome d ubiquinol oxidase subunit II (EC 1.10.3.-)	0.0001	2.1
GBAA5095	YP_021745.1	2-keto-3-deoxygluconate permease	0.0003	2.0
GBAA5097	YP_021747.1	2-dehydro-3-deoxygluconokinase (EC 2.7.1.45)	0.0001	2.2
GBAA5098	YP_021748.1	4-Hydroxy-2-oxoglutarate aldolase (EC 4.1.3.16) / 2-dehydro-3-deoxyphospho-gluconate aldolase (EC 4.1.2.14)	0.0004	2.1
GBAA5179	YP_021834.1	Hypothetical cytosolic protein	<0.0001	2.4
GBAA5237	YP_021889.1	Hypothetical lipoprotein	<0.0001	2.4
GBAA5239	YP_021891.1	regulatory protein (pfoS/R)	0.0024	2.2
GBAA5240	YP_021893.1	L-lactate dehydrogenase (EC 1.1.1.27)	<0.0001	2.2
GBAA5286	YP_021943.1	Na+/H+ antiporter napA	0.0017	2.6
GBAA5321	YP_021979.1	Hypothetical protein	0.0062	2.3
GBAA5322	YP_021980.1	Hypothetical protein	0.0017	3.0
GBAA5422	YP_022085.1	Ribosome-associated factor Y	<0.0001	2.3
GBAA5501	YP_022168.1	ABC transporter substrate-binding protein	0.0003	2.1
GBAA5605	YP_022276.1	Hypothetical protein	0.0067	3.3

This table reports genes with a significant difference in expression ($p<0.01$) between environments, as well as increased expression in CO_2 environments as compared to air (fold-change > 2).

Supplementary Table 9.
Genes having increased expression in environments of air (total = 219)

Locus Tag	Protein Product (NCBI)	Protein Annotation	P-Value	Fold-Change
GBAA_pXO1_0005	YP_016336.2	Hypothetical protein	<0.0001	2.9
GBAA_pXO1_0006	YP_016337.2	Hypothetical protein	0.0002	2.4
GBAA_pXO1_0018	YP_016349.2	Hypothetical protein	0.0018	3.0
GBAA_pXO1_0114	YP_016445.2	Hypothetical cytosolic protein	0.0071	2.3
GBAA0232	YP_016838.2	Oligopeptide transport system permease protein appB	0.0018	4.9
GBAA0325	YP_016941.1	4-aminobutyrate aminotransferase (EC 2.6.1.19)	0.0001	4.5
GBAA0364	YP_016983.1	Benzoate transport protein	0.0024	3.0
GBAA0381	YP_017001.1	Hydroxymethylpyrimidine transport system permease protein	0.0022	3.8
GBAA0387	YP_017007.1	Thioredoxin	0.0002	3.6
GBAA0412	YP_017035.1	Hypothetical Membrane Spanning Protein	0.007	3.2
GBAA0537	YP_017157.2	Peroxide operon regulator	<0.0001	7.9
GBAA0543	YP_017165.1	Monofunctional biosynthetic peptidoglycan transglycosylase (EC 2.4.2.-)	0.0017	3.1
GBAA0571	YP_017194.1	Two-component response regulator	<0.0001	3.9
GBAA0574	YP_017197.2	Hypothetical Protein	<0.0001	10.6
GBAA0587	YP_017211.1	Acetyltransferase, GNAT family (EC 2.3.1.-)	<0.0001	12.6
GBAA0592	YP_017218.1	Alanine dehydrogenase (EC 1.4.1.1)	0.0095	2.9
GBAA0631	YP_017259.1	PTS system, trehalose-specific IIBC component (EC 2.7.1.69)	<0.0001	88.8
GBAA0675	YP_017304.1	(R,R)-butanediol dehydrogenase (EC 1.1.1.4)	<0.0001	5.5
GBAA0689	YP_017318.1	Di-/tripeptide transporter	<0.0001	12.3
GBAA0739	YP_017371.1	Potassium-transporting ATPase A chain (EC 3.6.3.12)	<0.0001	15.7
GBAA0740	YP_017372.1	Potassium-transporting ATPase B chain (EC 3.6.3.12)	<0.0001	18.0
GBAA0741	YP_017373.1	Potassium-transporting ATPase C chain (EC 3.6.3.12)	<0.0001	15.2
GBAA0742	YP_017374.2	Sensor protein kdpD (EC 2.7.13.3)	<0.0001	10.6
GBAA0745	YP_017377.1	Phospholipase (EC 3.1.-.-)	<0.0001	8.1
GBAA0766	YP_017399.1	Nitroreductase family	0.0015	4.7
GBAA0796	YP_017431.1	SH3 domain protein / 3D domain protein	0.0096	4.6
GBAA0797	YP_017432.2	ABC transporter permease protein	<0.0001	4.9
GBAA0798	YP_017433.1	ABC transporter ATP-binding protein	<0.0001	6.4
GBAA0799	YP_017434.1	HlyD family secretion protein	<0.0001	6.7
GBAA0829	YP_017467.1	Transporter, Drug/Metabolite Exporter family	<0.0001	20.2

GBAA0855	YP_017492.1	Cystine-binding protein	0.0025	3.6
GBAA0856	YP_017493.2	Cystine transport system permease protein	0.0035	3.0
GBAA0866	YP_017503.2	Acetolactate synthase (EC 2.2.1.6)	<0.0001	18.7
GBAA0867	YP_017504.1	Alpha-acetolactate decarboxylase (EC 4.1.1.5)	<0.0001	15.5
GBAA0871	YP_017508.1	Collagen adhesion protein	<0.0001	6.4
GBAA1032	YP_017659.2	RNA polymerase ECF-type sigma factor	<0.0001	7.0
GBAA1034	YP_017661.1	Rod shape-determining protein rodA	<0.0001	5.6
GBAA1158	YP_017774.1	Ferrochelatase (EC 4.99.1.1)	<0.0001	48.3
GBAA1159	YP_017775.1	Catalase (EC 1.11.1.6)	<0.0001	7.8
GBAA1169	YP_017783.1	Peptidyl-prolyl cis-trans isomerase (EC 5.2.1.8)	<0.0001	7.4
GBAA1174	YP_017788.1	Hydrolase (HAD superfamily)	0.007	2.9
GBAA1208	YP_017819.1	Hypothetical cytosolic protein	0.0072	2.7
GBAA1209	YP_017820.1	Globin family protein	0.001	3.4
GBAA1287	YP_017902.1	Signal peptidase I (EC 3.4.21.89)	<0.0001	12.4
GBAA1288	YP_017903.1	Hypothetical protein	<0.0001	8
GBAA1308	YP_017922.1	(S)-2-hydroxy-acid oxidase subunit GlcF (EC 1.1.3.15)	0.0005	3.8
GBAA1309	YP_017923.1	(S)-2-hydroxy-acid oxidase chain D (EC 1.1.3.15)	0.0001	4.2
GBAA1348	YP_017969.1	CBS domain containing protein	0.003	3.0
GBAA1467	YP_018089.1	Flavohemoprotein / Dihydropteridine reductase (EC 1.5.1.34) / Nitric oxide dioxygenase (EC 1.14.12.17)	<0.0001	3.8
GBAA1497	YP_018120.1	Transcriptional regulatory protein resD	0.0038	2.8
GBAA1605	YP_018228.1	similar to N-terminus of Mg(2+) P-type ATPase	0.0038	2.9
GBAA1636	YP_018271.2	Adenosylmethionine-8-amino-7-oxononanoate aminotransferase (EC 2.6.1.62)	0.0013	3.1
GBAA1751	YP_018392.1	Asparagine synthetase [glutamine-hydrolyzing] (EC 6.3.5.4)	<0.0001	13.1
GBAA1754	YP_018394.1	ECF-type sigma factor negative effector	0.0063	2.8
GBAA1756	YP_018396.1	Cobalt-zinc-cadmium resistance protein czcD	0.0002	3.4
GBAA1767	YP_018408.1	Aldose 1-dehydrogenase (EC 1.1.1.121) / Fumarate hydratase class II (EC 4.2.1.2)	0.0089	2.7
GBAA1774	YP_018413.1	Peptidase E (EC 3.4.11.2)	<0.0001	5.8
GBAA1785	YP_018424.2	Hypothetical Membrane Spanning Protein	0.0097	2.6
GBAA1793	YP_018431.1	Export ABC transporter ATP-binding protein	<0.0001	6.8
GBAA1794	YP_018432.1	Export ABC transporter permease protein	<0.0001	5.9
GBAA1795	YP_018433.1	Export ABC transporter permease protein	<0.0001	4.3
GBAA1796	YP_018434.1	Cardiolipin synthetase (EC 2.7.8.-)	<0.0001	3.9
GBAA1799	YP_018436.1	Proton/sodium-glutamate symport protein	<0.0001	12.8
GBAA1800	YP_018437.1	Aspartate ammonia-lyase (EC 4.3.1.1)	<0.0001	7.3

GBAA1801	YP_018438.1	NAD-dependent malic enzyme (EC 1.1.1.38)	0.0002	3.3
GBAA1862	YP_018502.1	Succinyl-CoA:coenzyme A transferase (EC 2.8.3.-)	<0.0001	35.8
GBAA1863	YP_018503.1	Acetyltransferase (EC 2.3.1.-)	0.002	3.4
GBAA1864	YP_018504.1	Hypothetical protein	0.0077	3.7
GBAA1875	YP_018516.1	Radical SAM superfamily protein	<0.0001	8.4
GBAA1880	YP_018522.1	Manganese transport protein mntH	0.0046	4.9
GBAA1968	YP_018612.1	Homoserine dehydrogenase (EC 1.1.1.3)	0.0064	2.8
GBAA1973	YP_018617.1	Transcriptional regulator, LytR family	0.0058	3.8
GBAA1974	YP_018618.1	Hypothetical protein	<0.0001	4.3
GBAA1975	YP_018619.1	Alkaline phosphatase synthesis two-component response regulator phoP	<0.0001	5.1
GBAA1980	YP_018624.2	Acetyltransferase (EC 2.3.1.-)	<0.0001	3.7
GBAA1981	YP_018625.1	Amino-acid citrate synthetase (EC 6.-.-.-)	<0.0001	3.6
GBAA2051	YP_018690.1	Hypothetical membrane spanning protein	<0.0001	5.0
GBAA2052	YP_018691.1	Hypothetical membrane spanning protein	<0.0001	3.8
GBAA2069	YP_018710.2	Acetyltransferase, GNAT family (EC 2.3.1.-)	0.0002	3.7
GBAA2070	YP_018711.1	D-alanyl-D-alanine metallocarboxypeptidase (EC 3.4.17.14)	<0.0001	3.9
GBAA2071	YP_018712.1	Phosphohydrolase (MutT/nudix family protein)	0.0001	3.5
GBAA2125	YP_018768.1	Respiratory nitrate reductase alpha chain (EC 1.7.99.4)	<0.0001	9.0
GBAA2126	YP_018769.1	Respiratory nitrate reductase beta chain (EC 1.7.99.4)	<0.0001	9.6
GBAA2127	YP_018770.1	Respiratory nitrate reductase delta chain (EC 1.7.99.4)	<0.0001	8.8
GBAA2128	YP_018771.1	Respiratory nitrate reductase gamma chain (EC 1.7.99.4)	0.0005	8.9
GBAA2133	YP_018776.2	Molybdenum cofactor biosynthesis protein A	<0.0001	12.7
GBAA2134	YP_018777.1	Molybdopterin biosynthesis MoeB protein	<0.0001	9.6
GBAA2135	YP_018778.1	Molybdopterin biosynthesis MoeA protein	<0.0001	7.7
GBAA2136	YP_018779.1	Molybdopterin converting factor, large subunit	<0.0001	9.7
GBAA2137	YP_018780.1	Molybdopterin converting factor, small subunit	<0.0001	12.3
GBAA2138	YP_018781.1	Nitrite extrusion protein	<0.0001	7.9
GBAA2143	YP_018786.1	Sirohydrochlorin cobaltochelatase (EC 4.99.1.3)	0.0033	2.8
GBAA2144	YP_018787.1	Uroporphyrin-III C-methyltransferase (EC 2.1.1.107) / Uroporphyrinogen-III synthase (EC 4.2.1.75)	0.0041	2.8
GBAA2145	YP_018788.1	Nitrite reductase [NAD(P)H] small subunit (EC 1.7.1.4)	0.0052	2.6
GBAA2147	YP_018790.1	Nitric oxide-dependent regulator DnrN	<0.0001	4.0
GBAA2255	YP_018900.1	Fe3+-siderophore binding protein	<0.0001	15.4

GBAA2261	YP_018906.1	Phosphohydrolase (MutT/nudix family protein)	<0.0001	5.6
GBAA2279	YP_018929.1	Glycine betaine transport ATP-binding protein	<0.0001	5.5
GBAA2280	YP_018930.1	Glycine betaine transport system permease protein / Glycine betaine-binding protein	0.0004	3.4
GBAA2289	YP_018938.1	Succinate-semialdehyde dehydrogenase [NADP+] (EC 1.2.1.16)	<0.0001	4.0
GBAA2317	YP_018965.1	5-methylcytosine-specific restriction enzyme MRR (EC 3.1.21.-)	0.0035	2.9
GBAA2366	YP_019010.1	Hypothetical protein	<0.0001	63.5
GBAA2367	YP_019011.1	Oxalate/formate antiporter	<0.0001	18.1
GBAA2388	YP_019030.1	Threonyl-tRNA synthetase (EC 6.1.1.3)	<0.0001	4.8
GBAA2390	YP_019033.1	ABC transporter permease protein	0.0069	8.5
GBAA2393	YP_019035.2	Macrolide-efflux protein	<0.0001	6.3
GBAA2443	YP_019084.1	Multidrug resistance ABC transporter ATP-binding and permease protein	0.0003	3.4
GBAA2444	YP_019085.1	Multidrug/protein/lipid ABC transporter family, ATP-binding and permease protein	<0.0001	3.8
GBAA2564	YP_019202.2	5'-methylthioadenosine nucleosidase (EC 3.2.2.16) / S-adenosylhomocysteine nucleosidase (EC 3.2.2.9)	<0.0001	3.8
GBAA2565	YP_019203.1	Putative NAD-dependent dehydrogenase	0.0009	3.2
GBAA2569	YP_019208.1	hypothetical protein	0.0038	3.2
GBAA2619	YP_022616.1	Hypothetical protein	<0.0001	6.3
GBAA2620	YP_019261.1	Hypothetical protein	<0.0001	4.0
GBAA2707	YP_019348.1	Acetyltransferase (EC 2.3.1.-)	0.0033	2.9
GBAA2783	YP_019425.2	Hypothetical protein	0.0089	3.1
GBAA2803	YP_019445.1	S-layer homology domain / Metallo-beta-lactamase superfamily	<0.0001	5.5
GBAA2809	YP_019450.1	Hypothetical Membrane Spanning Protein	0.001	3.3
GBAA2868	YP_019512.1	N-acetylglucosaminyldiphosphoundecaprenol N-acetyl-beta-D-mannosaminyltransferase (EC 2.4.1.187)	0.0069	2.8
GBAA2889	YP_019532.1	Hypothetical protein	0.0061	2.8
GBAA2947	YP_019588.1	Sulfatase family protein	<0.0001	9.8
GBAA2966	YP_019608.1	Putative kinase	0.0098	4.1
GBAA2990	YP_019633.1	Hypothetical protein	0.0022	3.1
GBAA3010	YP_019654.1	Acetyltransferase, GNAT family (EC 2.3.1.-)	0.0002	3.5
GBAA3020	YP_052630.1	Proline/betaine transporter	<0.0001	18.0
GBAA3076	YP_019717.1	Lysine-specific permease	<0.0001	5.5
GBAA3077	YP_019718.2	HesB-like protein	0.0001	4.1
GBAA3078	YP_019719.1	Hypothetical protein	0.0002	3.6
GBAA3100	YP_019742.1	Copper homeostasis protein cutC	0.0056	4.7
GBAA3137	YP_019776.1	L-asparaginase (EC 3.5.1.1)	0.004	2.8

GBAA3140	YP_019779.1	UvrC-like protein	0.0033	4.4
GBAA3141	YP_019780.2	Amino acid permease	<0.0001	4.5
GBAA3142	YP_019781.1	Branched chain amino acid transport system II carrier protein	<0.0001	4.4
GBAA3143	YP_019782.1	Pyrroline-5-carboxylate reductase (EC 1.5.1.2)	0.0095	3.8
GBAA3144	YP_019783.1	Hypothetical protein	0.0021	4.6
GBAA3145	YP_019784.1	NAD-dependent malic enzyme (EC 1.1.1.38)	<0.0001	5.4
GBAA3146	YP_022631.1	Hypothetical protein	0.0002	6.5
GBAA3147	YP_019785.1	Hypothetical protein	0.0031	5.7
GBAA3164	YP_019804.1	Catalase (EC 1.11.1.6)	<0.0001	4.2
GBAA3165	YP_019805.1	Arylesterase (EC 3.1.1.2)	<0.0001	3.7
GBAA3167	YP_019807.1	Hydrolase, isochorismatase family	0.0005	3.5
GBAA3168	YP_019808.1	hypothetical protein	0.0028	3.4
GBAA3180	YP_019820.1	Deoxyribodipyrimidine photolyase (EC 4.1.99.3)	0.0012	3.5
GBAA3182	YP_019823.1	hypothetical protein	0.002	4.1
GBAA3227	YP_019866.1	Hypothetical Protein	<0.0001	26.8
GBAA3228	YP_019867.1	Hypothetical cytosolic protein	0.0013	20.4
GBAA3253	YP_019890.1	Hypothetical protein	0.0021	10.6
GBAA3254	YP_019891.1	Surface protein	<0.0001	3.5
GBAA3255	YP_019892.1	ABC transporter ATP-binding protein	<0.0001	4.5
GBAA3256	YP_019893.1	ABC transporter permease protein	<0.0001	5.6
GBAA3275	YP_019910.2	Muramoyltetrapeptide carboxypeptidase (EC 3.4.17.13)	0.0002	3.5
GBAA3317	YP_019951.2	Amino acid permease	<0.0001	6.9
GBAA3323	YP_019957.1	Sigma-M negative effector	0.0001	4.1
GBAA3324	YP_019958.1	RNA polymerase sigma factor sigM	<0.0001	4.9
GBAA3338	YP_019972.1	S-layer protein	0.0057	2.8
GBAA3368	YP_020001.1	S-adenosylmethionine:2-demethylmenaquinone methyltransferase (EC 2.1.-.-)	0.0017	3.1
GBAA3473	YP_020108.1	Long-chain-fatty-acid--CoA ligase (EC 6.2.1.3)	<0.0001	15.4
GBAA3497	YP_020132.1	Hydroxylamine reductase (EC 1.7.99.1)	<0.0001	7.8
GBAA3498	YP_020133.1	Hypothetical protein	0.0031	3.1
GBAA3524	YP_020159.1	GlcNAc-PI de-N-acetylase family protein	0.0033	2.9
GBAA3538	YP_020172.1	Hypothetical protein	<0.0001	5.4
GBAA3554	YP_020187.1	Beta-lactamase family protein	<0.0001	6.5
GBAA3595	YP_020230.2	BNR-repeat containing protein	<0.0001	6.1
GBAA3603	YP_020238.2	Acetyltransferase (EC 2.3.1.-)	<0.0001	5.3
GBAA3609	YP_020244.1	Aldehyde dehydrogenase (EC 1.2.1.3)	0.0052	5.3
GBAA3628	YP_020261.2	Formate dehydrogenase accessory protein FdhD	0.0005	3.2

GBAA3647	YP_020281.1	Transcriptional regulator, LytR family	<0.0001	31.2
GBAA3648	YP_020282.1	ECF-type sigma factor negative effector	<0.0001	33.5
GBAA3649	YP_020283.1	RNA polymerase ECF-type sigma factor	<0.0001	33.9
GBAA3730	YP_020367.1	Murein hydrolase exporter	0.0004	5.5
GBAA3731	YP_020368.1	Murein hydrolase export regulator	<0.0001	5.1
GBAA3739	YP_020377.2	Hypothetical protein	0.0033	3.0
GBAA3852	YP_020489.1	Hypothetical protein	<0.0001	6.9
GBAA3866	YP_020504.1	Fe3+-siderophore transport system permease protein	0.0021	5.1
GBAA3896	YP_020534.1	Tetratricopeptide repeat family protein	<0.0001	7.6
GBAA3995	YP_020635.1	General stress protein, Gls24 family	0.0077	2.6
GBAA4189	YP_020833.1	Putative transcriptional regulator	<0.0001	3.7
GBAA4205	YP_020849.2	phosphoesterase (EC 3.1.-.-)	0.0098	2.8
GBAA4249	YP_020891.2	D-beta-hydroxybutyrate dehydrogenase (EC 1.1.1.30)	0.0031	2.9
GBAA4295	YP_020938.1	Anti-sigma F factor	0.0045	2.8
GBAA4369	YP_021013.1	Ribosomal-protein-S5-alanine acetyltransferase (EC 2.3.1.128)	0.0017	2.8
GBAA4499	YP_021143.1	Superoxide dismutase (EC 1.15.1.1)	0.0005	4.5
GBAA4569	YP_021214.1	Hypothetical cytosolic protein	0.0017	3.5
GBAA4595	YP_021241.1	Ferrichrome transport ATP-binding protein fhuC	0.0001	6.1
GBAA4596	YP_021242.1	Ferrichrome transport system permease protein fhuB	<0.0001	11.2
GBAA4597	YP_021243.1	Ferrichrome-binding protein	<0.0001	17.1
GBAA4652	YP_021299.1	Ferrichrome-binding protein	<0.0001	15.0
GBAA4666	YP_021313.1	Prephenate dehydratase (EC 4.2.1.51)	0.0069	2.6
GBAA4667	YP_021314.1	ABC transporter permease protein	<0.0001	6.4
GBAA4668	YP_021315.1	ABC transporter permease protein	<0.0001	31.1
GBAA4669	YP_021316.1	ABC transporter ATP-binding protein	<0.0001	26.2
GBAA4781	YP_021427.1	Na(+)/H(+) antiporter	<0.0001	5.6
GBAA4789	YP_021436.1	Cell surface protein	<0.0001	42.9
GBAA4807	YP_021452.1	Metal dependent phosphohydrolase	<0.0001	4.5
GBAA4808	YP_021453.1	CAAX amino terminal protease family	0.0006	6.5
GBAA4812	YP_021457.1	Multidrug resistance protein B	<0.0001	39.7
GBAA4813	YP_021459.1	Multidrug resistance protein A	<0.0001	50.8
GBAA5061	YP_021711.1	Phage shock protein A	0.0095	3.6
GBAA5062	YP_021712.1	Hypothetical protein	0.0003	3.9
GBAA5063	YP_022716.1	Hypothetical protein	<0.0001	11.1
GBAA5064	YP_021713.1	Ferrous iron transport protein B	<0.0001	20.8
GBAA5065	YP_021714.1	Ferrous iron transport protein A	<0.0001	35.7
GBAA5066	YP_021715.1	Thiamine transporter	0.0038	3.0
GBAA5084	YP_021732.1	ABC transporter permease protein	<0.0001	4.2
GBAA5085	YP_021733.1	ABC transporter ATP-binding protein	<0.0001	5.4

GBAA5256	YP_052653.1	Methyl-accepting chemotaxis protein	0.0012	2.7
GBAA5262	YP_021918.1	Hypothetical exported repetitive protein	<0.0001	15.8
GBAA5279	YP_021936.1	SAM-dependent methyltransferase	0.002	3.3
GBAA5290	YP_021946.1	Non-specific DNA-binding protein Dps / Iron-binding ferritin-like antioxidant protein / Ferroxidase (EC 1.16.3.1)	0.0014	2.9
GBAA5298	YP_021956.1	Nucleoside permease nupC	0.0014	3.0
GBAA5330	YP_021988.2	Ferric anguibactin-binding protein	0.0006	3.0
GBAA5405	YP_022068.2	Hypothetical membrane spanning protein	<0.0001	21.0
GBAA5427	YP_022089.2	NlpC/P60 family protein	0.0001	3.2
GBAA5443	YP_022106.1	PTS system, cellobiose-specific IIC component (EC 2.7.1.69)	0.0002	3.8
GBAA5481	YP_022147.1	SH3 domain protein / 3D domain protein	0.0028	3.9
GBAA5620	YP_022292.1	Transporter, MFS superfamily	0.0004	3.8
GBAA5630	YP_022304.1	Ferrichrome transport system permease protein fhuG	0.0049	3.1
GBAA5636	YP_022311.1	Phosphate acetyltransferase (EC 2.3.1.8)	0.0007	3.1
GBAA5689	YP_022370.1	Murein hydrolase export regulator	<0.0001	29.8
GBAA5690	YP_022372.1	Murein hydrolase exporter	0.0035	57.2

This table reports genes with a significant difference in expression ($p<0.01$) between environments, as well as increased expression in air environments as compared to CO_2 (fold-change > 2).

Supplementary Figure 1.
Representative growth curves of wildtype and ΔAtxA strains in air and CO_2

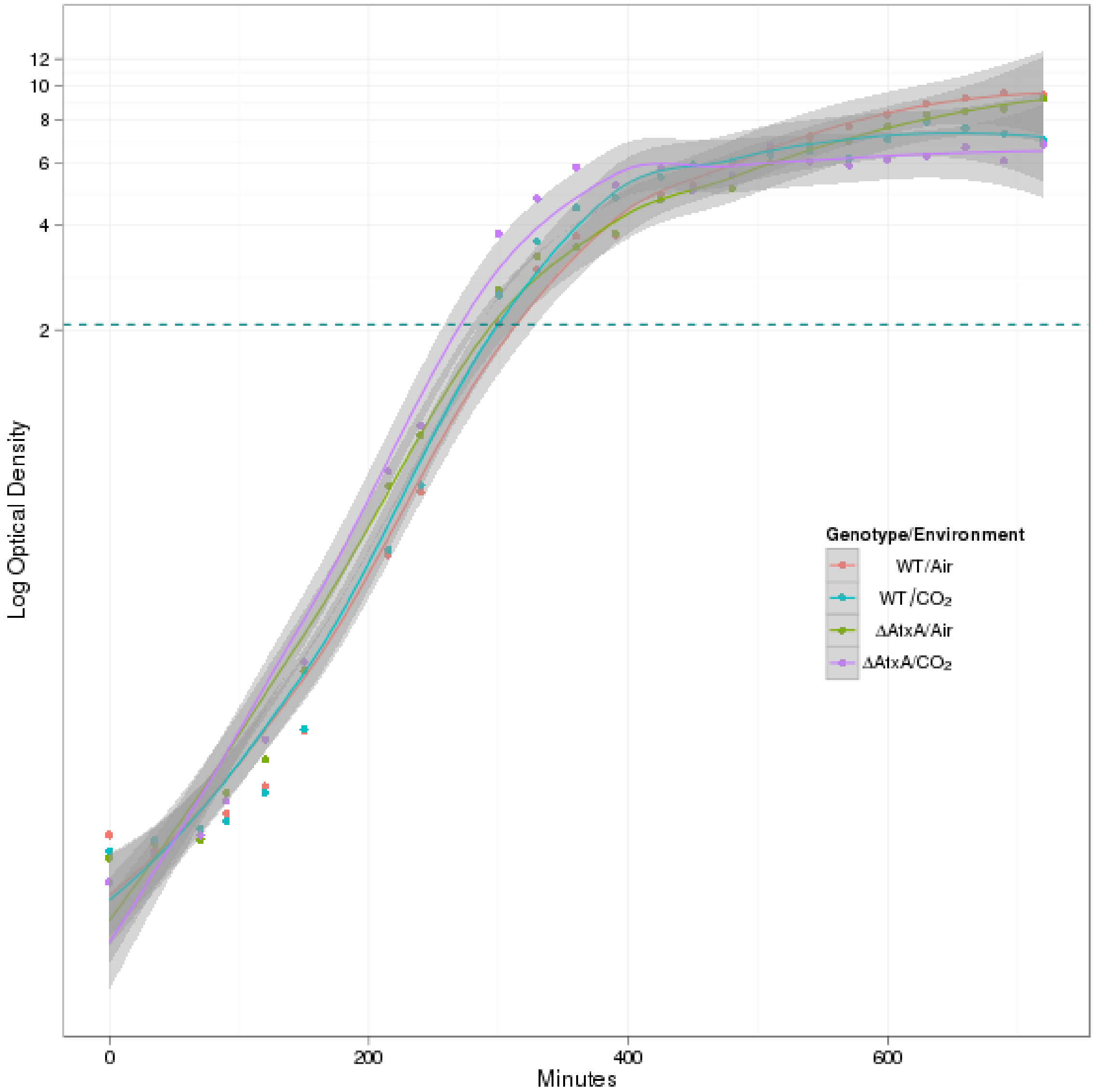

Growth curves for strains cultured in NBY media, where environments of CO_2 denote the addition of 15% CO_2 and 0.8% HCO_3. OD_{600nm} readings are transformed to a log base-10 scale. The data points for each curve are fit with a locally weighted scatterplot smoothing regression model, and the grey areas represent 95% confidence intervals. The dotted teal line indicates the mean OD_{600nm} at which we harvested our samples. This plot was made using the R package ggplot2.

Supplementary Figure 2: Comparison of RNA-seq to qPCR expression data of seven genes from each condition.

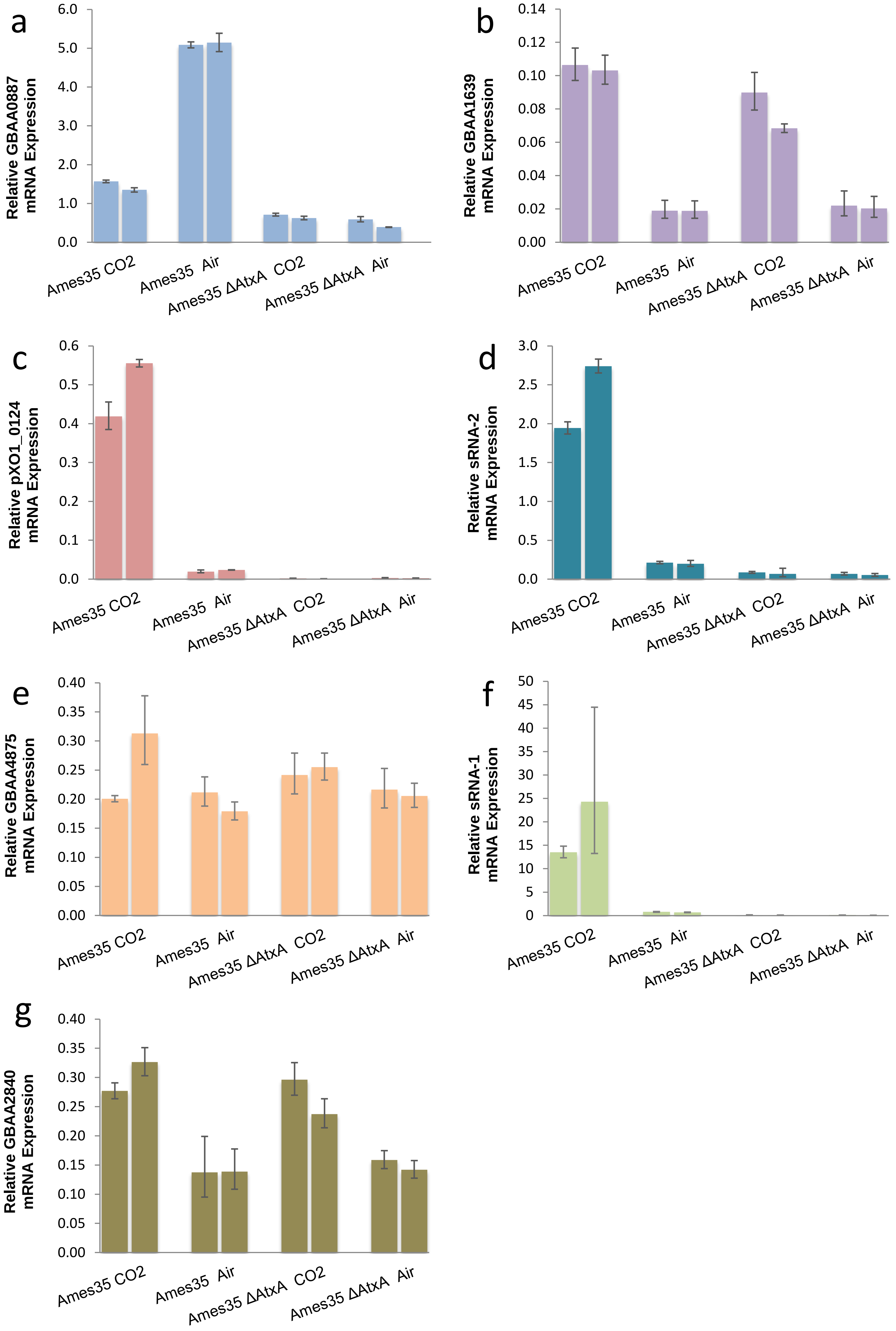

Expression levels for each genes from both sequencing modalities were normalized to the expression of GBAA3936, which codes for the house keeping protein aspartokinase. Genes are labeled by their locus tags (NCBI), except for sRNA-1 and sRNA-2, which are unannotated. The x-axis refers to the condition (of four) in which the RNA-seq and qPCR expression levels were assayed.
